# Ureidobenzenesulfonamides as Selective Carbonic Anhydrase I, IX, and XII Inhibitors

**DOI:** 10.3390/molecules28237782

**Published:** 2023-11-26

**Authors:** Toni C. Denner, Andrea Angeli, Marta Ferraroni, Claudiu T. Supuran, René Csuk

**Affiliations:** 1 Organic Chemistry, Martin-Luther University Halle-Wittenberg, Kurt-Mothes, Str. 2, D-06120 Halle (Saale), Germany; toni-christopher.denner@chemie.uni-halle.de; 2 Section of Pharmaceutical Sciences, Neurofarba Department, University of Florence, Via Ugo Schiff 6, Sesto Florentino, 50019 Florence, Italy; andrea.angeli@unifi.it (A.A.); claudiu.supuran@unifi.it (C.T.S.); 3 Department of Chemistry “Ugo Schiff”, University of Florence, Via della Lastruccia 3-13, Sesto Fiorentino, 50019 Florence, Italy; marta.ferraroni@unifi.it

**Keywords:** carbonic anhydrase, inhibitor, ureidobenzenesulfonamides

## Abstract

Sulfonamides remain an important class of drugs, especially because of their inhibitory effects on carbonic anhydrases. Herein, we have synthesized several sulfonamides and tested them for their inhibitory activity against carbonic anhydrases hCA I, hCA II, hCA IX, and hCA XII, respectively. Thereby, biphenyl- and benzylphenyl-substituted sulfonamides showed high selectivity against hCA IX and hCA XII; these enzymes are common targets in the treatment of hypoxic cancers, and noteworthy inhibitory activity was observed for several compounds toward hCA I that might be of interest for future applications to treat cerebral edema. Compound **3** (4-[3-(2-benzylphenyl)ureido]benzenesulfonamide) held an exceptionally low K_i_ value of 1.0 nM for hCA XII.

## 1. Introduction

The development of sulfonamides began as early as 1908 [1], when the antibacterial effect of “Prontosil” (4-(2,4-diaminophenyl)diazinyl)-benzene sulfonamide [2] was first successfully used to treat bacterial sepsis in humans [3]. While today, sulfonamides have—more or less—lost their importance as antibacterial drugs due to the development of other drug classes, a new era began with the observation that representatives of this substance class are excellent inhibitors of the enzyme carbonic anhydrase [4].

Carbonic anhydrases (CAs; EC 4.2.1.1) are essential for life, as they balance acid and base equilibria in tissues and blood through the conversion of carbon dioxide and water into bicarbonate and protons. The importance of CAs can be seen from their high turn-over numbers [5], which are even faster than those measured for the enzyme acetylcholinesterase (AChE), being necessary for synaptic transmission and hence belonging to the fastest catalyzing enzymes. Furthermore, it has been shown that an isoform, carbonic anhydrase IX, is overexpressed in many types of cancer, thereby leading to an acidosis of the surrounding tissue and, consequently, promoting tumor growth, invasion, and proliferation [6]. In addition, changes in the tumor microenvironment that are induced by hypoxia promote aggressive and resistant cancer phenotypes [7], thus resulting in a poor prognosis in cancer patients [8]. Especially in recent years, the development of carbonic anhydrase inhibitors (CAIs) is of major interest [9,10], as CAIs may be supportive in anticancer therapy [11]. Especially, targeting hCA IX and XII seems to be of major interest, as these enzymes are overexpressed in hypoxic tumors including breast, cervix, and lung carcinomas [12,13,14,15,16,17].

These metalloenzymes play a role in numerous physiological and pathological processes. Two out of the fifteen human CA isoforms, namely, hCA IX and XII, are heavily expressed in hypoxic tumors due to the activation of HIF-1/2 (the transcription factor hypoxia inducible factor). CA XII is moderately present, while CA IX is almost nonexistent in normal tissues. This renders them highly promising prospects for developing innovative anticancer drugs. Thus, HIF-1 is associated with tumorigenesis and other physiological or pathological processes, along with biochemical mechanisms that are responsible for these phenomena. Indeed, in hypoxia and particularly in hypoxic tumors, numerous proteins that are implicated in metabolism, pH regulations, angiogenesis, and immunological response are overexpressed due to the HIF signal cascade, rendering these tumor cells rather distinct from their normal counterparts.

However, the application of sulfonamide-based inhibitors acting on all different isoforms of CA may result in severe side effects like paresthesias and others, and especially in patients with pre-existing renal, pulmonary, and hepatic diseases, the most important concerns are acidosis, respiratory failure, and encephalopathy [15]. Consequently, isoform selectivity is of the utmost importance in order to avoid or minimize undesired side effects.

**Figure 1:**
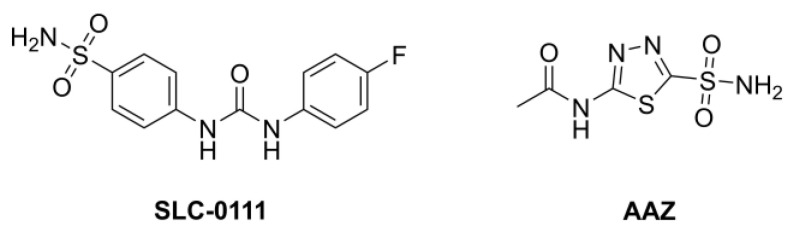
Structure of **SLC-0111** and of acetalzolamide (**AAZ**).

Several sulfonamides have already been synthesized and extensively investigated for their potential as inhibitors of CAs, as they appear to be very promising agents for the treatment of hypoxic tumors, especially for inhibiting the CA isoforms IX and XII since they are in vivo under the control of HIF-1, the transcription factor hypoxia-inducible factor. In particular, SLC-0111 (Figure 1), a sulfonamide inhibitor in clinical trials, seems to be very promising [18,19,20]. This compound is used in Phase 1b/II for combination treatment together with gemcitabine of metastatic pancreatic ductal adenocarcinoma.

Thus, HIF-1 is associated with tumorigenesis and other physiological or pathological processes, along with biochemical mechanisms that are responsible for these phenomena. Indeed, in hypoxia and particularly in hypoxic tumors, numerous proteins that are implicated in metabolism, pH regulations, angiogenesis, and immunological response are overexpressed due to the HIF signal cascade, rendering these tumor cells rather distinct from their normal counterparts. In response to hypoxia, tumor cells also experience transcriptional reprogramming involving increased expression of hCA IX, which maintains the acid–base balance. An overexpression of CA IX has been demonstrated to be associated with a negative prognosis in multiple cancers.

Furthermore, **SLC-0111** administration negated the hypoxia-triggered overexpression of CA IX and reduced cell viability, and **SLC-0111** also hindered cell migration in a wound healing experiment. In addition, transcriptomic profiles exhibited contrasting responses to **SLC-0111** administration between normoxic and hypoxic conditions, despite eliciting upregulation of various tumor suppressor genes in both scenarios.

**SLC-0111** holds an ureido moiety that is found in several compounds used in a broad variety of biomedical applications. Recently, some ureidobenzenesulfonamides have been reported as efficient inhibitors of carbonic anhydrase II [21]. The inhibition of this enzyme is essential in order to reduce intraocular pressure in patients suffering from glaucoma; it is expected that in 2040, the number of persons suffering from glaucoma will increase to 111.8 million [22]. However, the treatment of primary open-angle glaucoma, a multifactorial optic neuropathy that is linked to progressive retinal ganglion cell death and visual field loss, is one of the primary uses of CA II inhibitors. Elevated intraocular pressure is the primary risk factor for primary open-angle glaucoma, although there are numerous other related variables. Because of the unsatisfactory side effect profile of oral carbonic anhydrase inhibitors, topical CAIs took a long time to develop but have been shown to be a valuable adjunct to the treatment of primary open-angle glaucoma. They work by preventing the ciliary epithelium’s CA II from functioning. As a result, fewer bicarbonate ions are formed, which decreases intraocular pressure and fluid transfer.

Furthermore, out of all the organs studied, the mammalian central nervous system (CNS) possesses the greatest number of CA isoforms (at least 9). Isoforms I, VB, VII, VIII, X, XI, XII, and XIV are also found, with hCA II being the most prevalent. Carbonic anhydrase inhibitors have been used therapeutically in a number of brain pathologies because of the broad expression range of CA isoforms in the brain. In epilepsy and idiopathic intracranial hypertension, where acetazolamide (**AAZ**, Figure 1) is one of the medications now in clinical use, inhibition via CAIs has been shown to be clinically beneficial. Moreover, migraine, neuropathic pain, diabetes-induced blood–brain barrier failure, and amyloid β-induced mitochondrial dysfunction that is characteristic of Alzheimer’s disease are possible therapeutic uses for CAIs targeting CNS isoforms.

Consequently, the treatment of cerebral edema became a subject of scientific interest again, since swelling of the brain is a known side effect of some new drugs (e.g., lecanemab [23] and donanemab [24,25]) that were recently approved for the therapy of Alzheimer’s disease. Treatment of cerebral edema might include the intravenous injection of a CA inhibitor, especially of an inhibitor acting on hCA I [26,27].

## 2. Results

**Scheme 1:**
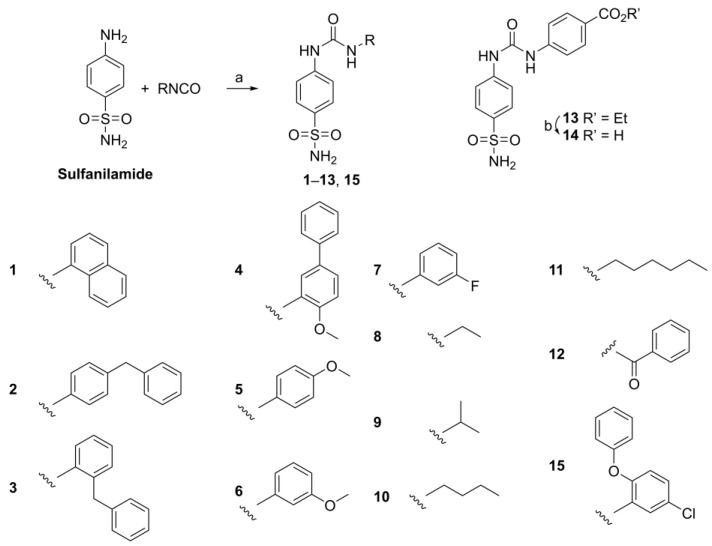
Synthesis of compounds **1**–**15**: (a) MeCN, 12 h, 23 °C: **1** naphthalen-1-yl, **2** 4-benzylphenyl, **3** 2-benzylphenyl, **4** 4-methoxy-(1,1′-biphenyl)-3-yl, **5** 4-methoxyphenyl, **6** 3-methoxyphenyl, **7** 3-fluorophenyl, **8** ethyl, **9** isopropyl, **10** butyl, **11** hexyl, **12** benzoyl, **13** ethyl-4-benzoate, **15** 5-chloro-2-phenoxyphenyl; (b) KOH, MeOH, ∆, 4 h, 98%.

Starting from a readily available starting material, compounds **1**–**15** were synthesized (Scheme 1) as previously described via the reaction of sulfanilamide with isocyanates [21]. This approach is especially suited for accessing the target compounds when the isocyanate is readily available. Recently, an alternative one-pot procedure has been published [28].

Furthermore, the ureido moieties incorporated into benzenesulfonamides holding an extra ureido moiety can be regarded as an interesting class of CA inhibitors (CAIs), since the presence of a urea functionality in the zinc-binding group is among the most promising trends in the design of CAIs.

In this study, we were especially interested in the investigation of benzylphenyl and biphenyl derivates, since previous docking studies indicated high binding affinities, which parallels previous results from the literature [21].

Compounds **1**–**15** were obtained in good yields and subjected to screening through stop–flow experiments employing enzymes hCA I, hCVA II, hCA IX, and hCA XII, respectively. Acetazolamide (**AAZ**, Figure 1) was used as a positive standard. The results from these assays are summarized in Table 1; data for **SLC-0111** were taken from the literature for comparison [29].

As a result of these assays, most of the compounds showed inhibition for all isoenzymes.

**Figure 2:**
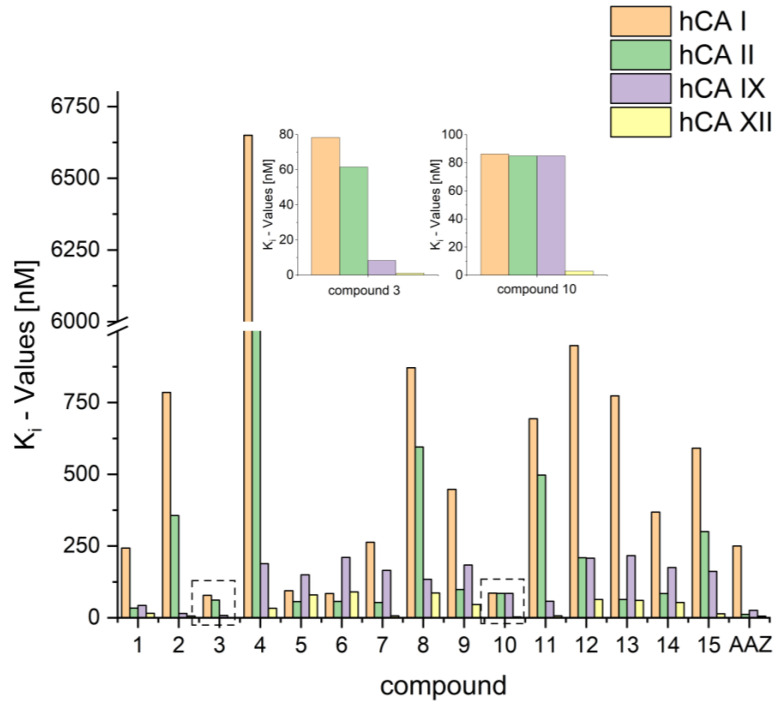
Depiction of K_i_ values for compounds **1**–**15** and **AAZ**.

However, it must be noted that compounds **2** and **3** exerted promising K_i_ values for hCA XII (K_i_ = 6.4 and 1.0 nm, respectively), with 3 being also an excellent inhibitor for hCA IX (K_i_ = 8.2 nm). A graphic comparison of all compounds is depicted in Figure 2.

**Figure 3:**
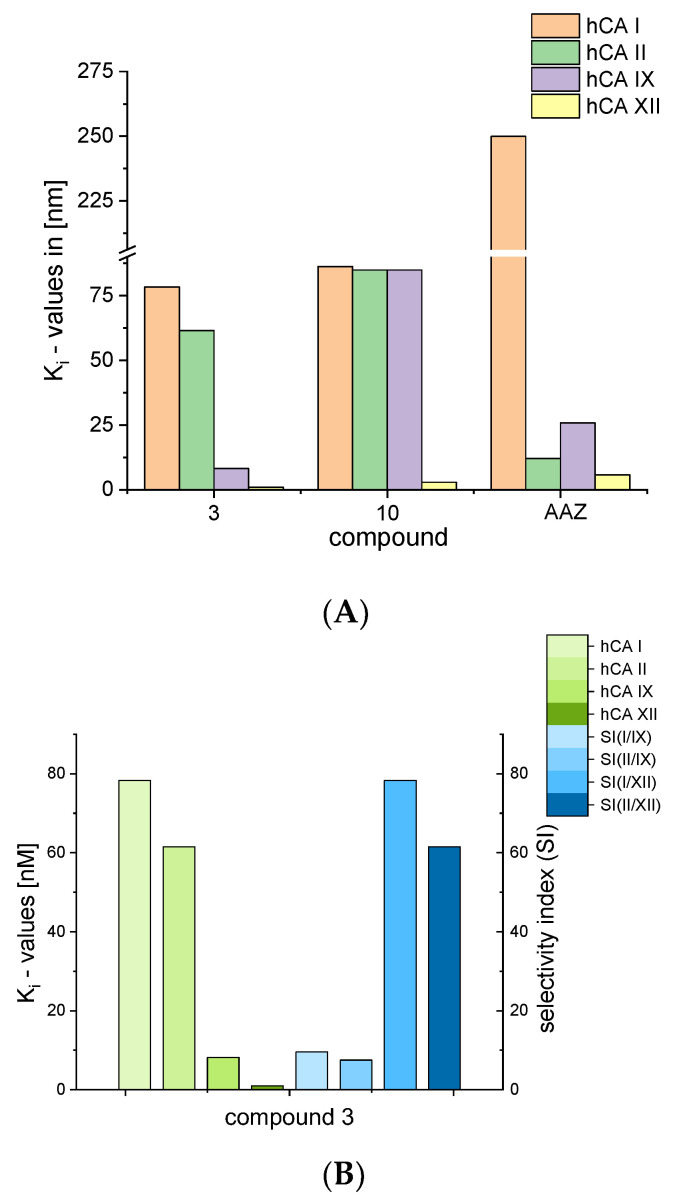
(**A**): Comparison of K_i_ values for compounds **3** and **10** and standard acetazolamide (**AAZ**); (**B**): comparison of K_i_ values for compound **3** with respect to isoforms of hCA.

Compound **3** exerted significantly lower K_i_ values for hCA I (compared with standard **AAZ**) and proved to be a better inhibitor of hCA IX and hCA XII than **AAZ** (Figure 3A). A comparison of the K_i_ values for compound **3** together with its isoform selectivity is depicted in Figure 3B.

**Figure 4:**
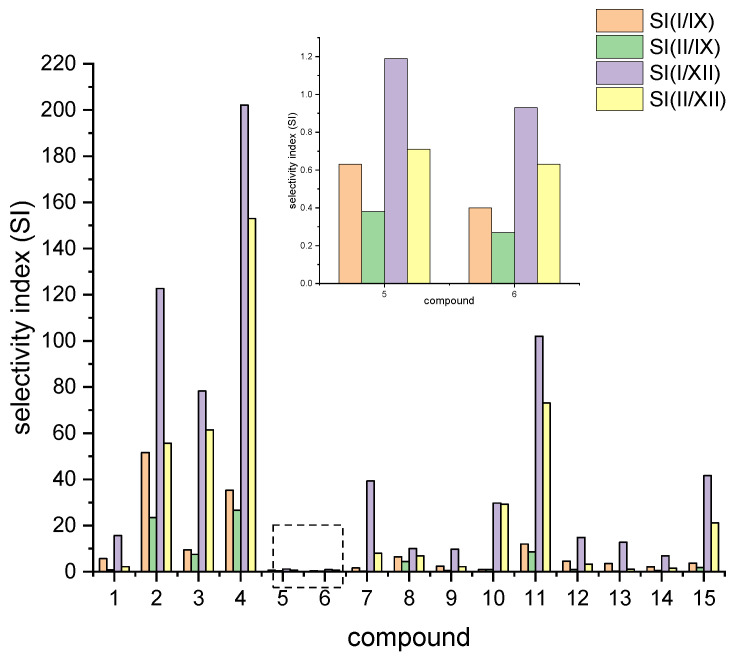
Comparison of selectivity index for compounds **1**–**15** with respect to the different isoforms.

Compound **4** is not a good inhibitor of both hCA I and hCA II, with K_i_ values > 5 µM. However, with K_i_ values of 188 nM and 32.9 nM for hCA IX and hCA XII, respectively, compound **4** holds a noteworthy selectivity, especially for hCA XII, with selectivity factors of 153 to 202, respectively, when compared with hCA I and hCA II. A comparison of the selective index is depicted in Figure 4.

While the biphenyl motif proved to be a very weak binder to hCA I and hCAII, the phenylbenzyl substituted ureas were good inhibitors of all tested isoenzymes and exhibited extraordinarily low K_i_ values for hCA IX and hCA XII (15 nM and 6.4 nM for compound **2**). Compound **3** even showed lower K_i_ values than **AZZ** with 8.2 nM against hCA IX and 1 nM against hCA XII. The low K_i_ values for compounds **2** and **3** also resulted in good selectivity factors of 23 to 122 for compound **2**. Compound **3**, however, held no pronounced selectivity for hCA IX but high selectivity of 78 and 62 for hCA XII.

**Figure 5:**
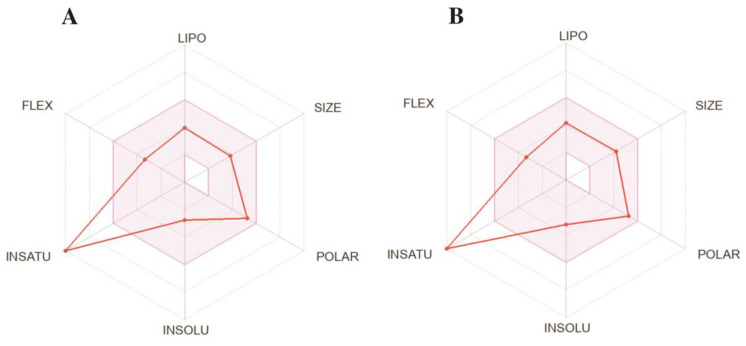
Predicted ADME properties of clinically tested SLC-0111 (**A**) and compound **3** (**B**).

The compound **SLC-0111** (Figure 1, K_i_ = 454 nM for hCA XII), which is currently used in clinical studies, was the best inhibitor of this study; compound **3**, regarding hCA XII (K_i_ = 1.0 nM), showed similar ADME profiles (Figure 5; calculated online www.swissadme.ch; accessed on 1 July 2023); this makes this compound an interesting candidate for further biological studies. The predicted ADME data for all compounds are compiled in the Supplementary Materials file.

As mentioned above, treatment of cerebral edema usually includes the application of a CA inhibitor. It might be assumed that a selective hCA I inhibitor might be beneficial for the therapy of this serious and life-threatening disease. Upon close examination of our results, a striking inhibitory disparity between compounds **8** and **10** became evident, approaching nearly 10-fold (with K_i_ values of 871.6 and 86.2 nM, respectively). Intriguingly, these compounds displayed minimal scaffolding differences, underscoring the pivotal role of subtle alkyl chain structural variances in dictating their divergent inhibitory activities against the targeted isoform. To unravel the molecular basis of these differences, X-ray diffraction experiments against hCA I were called for in order to obtain an insight into the ligand–protein interactions at the atomic level and focus on the tail interaction that underpinned the observed divergent potencies.

**Figure 6:**
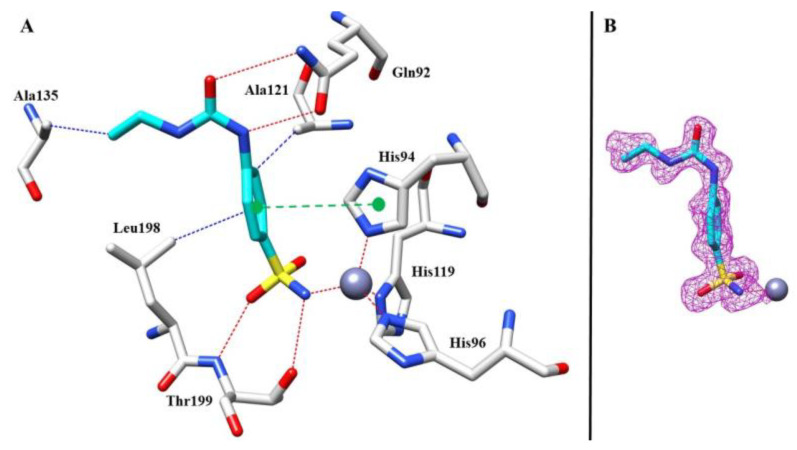
(**A**) Compound **8** inside the active site of hCA I. Hydrophobic (blue), hydrophilic (red), and π-stacking (green) interactions are labeled. (**B**) Electron density 2Fo-Fc map of **8** bound to zinc in hCA I active site; contoured at the 1.0 σ level.

Regarding the complex between **8** and hCA I, after the initial rounds of refinement, the calculated Fo-Fc map showed, inside the active site, a clear electron density that was compatible with the sulfonamide moiety (Figure 6B), which interacts directly with the zinc atom in the active sites. In addition, the sulfonamide group formed the characteristic hydrogen bond with residue Thr199, stabilizing the complex, which is typical of this class of inhibitors (Figure 6A).

Other hydrogen bonds were observed for the ureido moiety with a side chain of Gln92, and the benzene ring formed a π-stacking interaction with the aromatic ring of His94. Finally, we observed hydrophobic connections between Leu198 and Ala121 with the benzenesulfonamide moiety and between Ala135 and the end of the ethyl tail.

**Figure 7:**
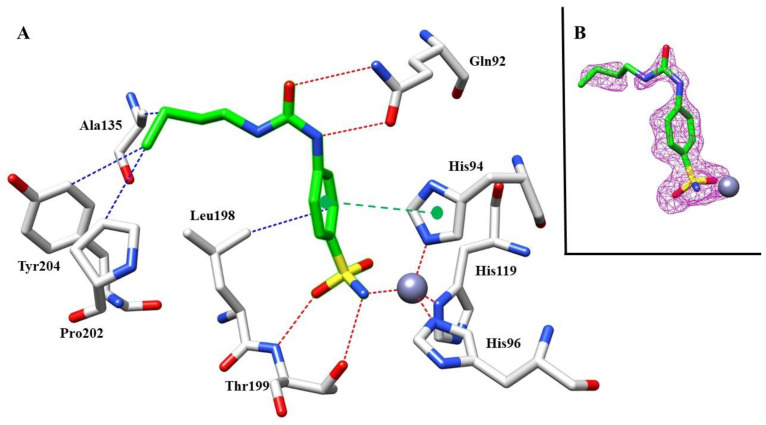
(**A**) Compound **10** inside the active site of hCA I. Hydrophobic (blue), hydrophilic (red), and π-stacking (green) interactions are labeled. (**B**) Electron density 2Fo-Fc map of **10** bound to zinc in hCA I active site; contoured at the 1.0 σ level.

Moving on the second complex investigated, 10 with hCA I, we also found a clear density for the sulfonamide moiety inside the active site of the protein (Figure 7B). The benzenesulfonamide scaffold showed the same binding mode as mentioned above, with the hydrogen bonds between the sulfonamide group and Thr199 stabilizing the binding of the inhibitor, similar to the previous complex with hCA I (Figure 7A).

Although a structural comparison of the two inhibitors with hCA I showed similar features, such as the same sulfonamide moiety interacting with the catalytic zinc ion and hydrogen bonds with ureido group and π-stacking interactions, the longer butyl chain of **10** compared with the ethyl one of **8** allows for more hydrophobic interactions with the side chains of Pro202 and Tyr204. This simple modification could explain the different inhibition potency against hCA I of nearly 10-fold between **8** and **10**.

## 3. Experimental Procedure

Reagents were bought from commercial suppliers and used without further purification. The solvents were dried according to usual procedures. TLC was performed on silica gel (Macherey-Nagel (Macherey Nagel GmbH, Düren, Germany), detection with UV absorption). Melting points are uncorrected (Büchi M-565). NMR spectra were recorded using VARIAN spectrometers (Varian GmbH, Darmstadt, Germany) at 27 °C (δ given in ppm; *J* in Hz, typical experiments for assignments: ^13^C APT, HMBC, HSQC). ASAP-MS spectra were taken on an Advion expression CMS-L (Advion Interchim, Ithaca, NY, USA) with an ASAP/APCI Ion source (capillary voltage 150 V, capillary temperature 220 °C, and voltage of the ion source: 15 V; APCI source temperature 300 °C with 5 μA). IR spectra were recorded on a Perkin-Elmer Spectrum Two (UATR Two Unit; Perkin-Elmer GmbH, Rodgau, Germany. CA inhibition assays were performed as previously described.

### 3.1. General Procedure for the Synthesis of Compounds ***1**–**13*** (GP)

A solution of sulfanilamide (2.5 mmol, 431 mg) and the corresponding isocyanate (1 eq.) in dry acetonitrile (10 mL) was stirred for 12 h at 23 °C, and the precipitate was filtered off and subsequently washed with acetonitrile, ethanol, and diethyl ether (5 mL each), and dried in vacuo to afford **1**–**13** each as a colorless crystalline solid.

#### 3.1.1. 4-[3-(Naphthalen-1-yl)ureido]benzenesulfonamide (**1**)

Following GP, **1** was obtained in 72% yield; R_f_ = 0.54 (silica gel, CHCl_3_/DMF/MeOH, 16:1:1); m.p.: 252–254 °C (lit.: [21] 254 °C); MS (APCI): *m*/*z* (%) = 342.1 ([M+H]^+^, 88).

#### 3.1.2. 4-[3-(4-Benzylphenyl)ureido]benzenesulfonamide (**2**)

Following GP, **2** was obtained in 48% yield; R_f_ = 0.50 (silica gel, CHCl_3_/DMF/MeOH, 16:1:1); m.p.: 230–232 °C (lit: [21] 231 °C); MS (APCI): *m*/*z* (%) = 382.1 ([M+H]^+^, 40).

#### 3.1.3. 4-[3-(2-Benzylphenyl)ureido])benzenesulfonamide (**3**)

Following GP, **3** was obtained in 45% yield; R_f_ = 0.59 (silica gel, CHCl_3_/DMF/MeOH, 16:1:1); m.p.: 219–221 °C (lit.: [21] 220 °C); MS (APCI): *m*/*z* (%) = 382.1 ([M+H]^+^, 31).

#### 3.1.4. 4-{3-[(4-Methoxy-(1,1′-biphenyl)-3-yl]ureido}benzenesulfonamide (**4**)

Following GP, **4** was obtained in 42% yield; R_f_ = 0.53 (silica gel, CHCl_3_/DMF/MeOH, 16:1:1); m.p.: 271–272 °C (lit.: [21] 270 °C); MS (APCI): *m*/*z* (%) = 398.2 ([M+H]^+^, 25).

#### 3.1.5. 4-[3-(4-Methoxyphenyl)ureido])benzenesulfonamide (**5**)

Following GP, **5** was obtained in 65% yield; R_f_ = 0.45 (silica gel, CHCl_3_/DMF/MeOH, 16:1:1); m.p.: 227–229 °C (lit.: [21] 229 °C); MS (APCI): *m*/*z* (%) = 322.4 ([M+H]^+^, 18).

#### 3.1.6. 4-[3-(3-Methoxyphenyl)ureido]benzenesulfonamide (**6**)

Following GP, **6** was obtained in 53% yield; R_f_ = 0.45 (silica gel, CHCl_3_/DMF/MeOH, 16:1:1); m.p.: 238–239 °C (lit.: [21] 238 °C); MS (APCI): *m*/*z* (%) = 322.1 ([M+H]^+^, 17).

#### 3.1.7. 4-[3-(3-Fluorophenyl)ureido]benzenesulfonamide (**7**)

Following GP, **7** was obtained in 70% yield; R_f_ = 0.42 (silica gel, CHCl_3_/DMF/MeOH, 16:1:1); m.p.: 240–241 °C (lit.: [21] 240 °C); MS (APCI): *m*/*z* (%) = 310.3 ([M+H]^+^, 97).

#### 3.1.8. 4-(3-Ethylureido)benzenesulfonamide (**8**)

Following GP, **8** was obtained in 68% yield; R_f_ = 0.37 (silica gel, CHCl_3_/DMF/MeOH, 16:1:1); m.p.: 229–231 °C (lit.: [21] 232 °C); MS (APCI): *m*/*z* (%) = 244.0 ([M+H]^+^, 35).

#### 3.1.9. 4-(3-Isopropylureido)benzenesulfonamide (**9**)

Following GP, **9** was obtained in 54% yield; R_f_: = 0.65 (silica gel, CHCl_3_/DMF/MeOH, 16:1:1); m.p.: 221–223 °C (lit.: 223 °C); MS (APCI): *m*/*z* (%) = 256.3 ([M+H]^+^, 96).

#### 3.1.10. 4-(3-Butylureido)benzenesulfonamide (**10**)

Following GP, **10** was obtained in 68% yield; R_f_ = 0.43 (silica gel, CHCl_3_/DMF/MeOH, 16:1:1); m.p.: 194–196 °C (lit.: [21] 195 °C); MS (APCI): *m*/*z* (%) = 272.3 ([M+H]^+^, 25).

#### 3.1.11. 4-(3-Hexylureido)benzenesulfonamide (**11**)

Following GP, **11** was obtained in 71% yield; R_f_ = 0.49 (silica gel, CHCl_3_/DMF/MeOH, 16:1:1); m.p.: 202–203 °C (lit.: [21] 201 °C); MS (APCI): *m*/*z* (%) = 300.3 ([M+H^+^], 35).

#### 3.1.12. N-[(4-Sulfamoylphenyl)carbamoyl]benzamide (**12**)

Following GP, **12** was obtained in 51% yield; R_f_ = 0.71 (silica gel, CHCl_3_/DMF/MeOH, 16:1:1); m.p.: 262–264 °C (lit.: [21] 263 °C); MS (APCI): *m*/*z* (%) = 320.5 ([M+H]^+^, 11).

#### 3.1.13. Ethyl 4-[3-(4-sulfamoylphenyl)ureido]benzoate (**13**)

Following GP, **13** was obtained in 91% yield; R_f_ = 0.35 (silica gel, CHCl_3_/DMF/MeOH, 16:1:1); m.p.: 261–263 °C (lit.: [21] 263 °C); MS (APCI): *m*/*z* (%) = 364.3 ([M+H]^+^, 23).

#### 3.1.14. 4-[3-(4-Sulfamoylphenyl)ureido]benzoic acid (**14**)

To a suspension of **13** (200 mg, 0.55 mmol) in MeOH (10 mL), finely grounded KOH (310 mg, 5.5 mmol) was added, and the mixture was heated under reflux for 4 h. The precipitate was filtered off, washed with MeOH, and dried to afford **14** (196 mg, 98%) as a colorless solid; R_f_ = 0.10 (silica gel, CHCl_3_/DMF/MeOH, 16:1:1); m.p. 310–313 °C (lit.: [21] 277–303 °C); MS (APCI): *m*/*z* (%) = 336.4 ([M+H]^+^, 97).

#### 3.1.15. 4-[3-(5-Chloro-2-phenoxyphenyl)ureido]benzenesulfonamide (**15**)

To a solution of sulfanilamide (206 mg, 1.2 mmol) in acetonitrile (10 mL), 5-chloro-2-phenoxyphenylisocyanate (295 mg, 1.2 mmol) was added, and the mixture was stirred at 21 °C for 1 day. The volatiles were removed under reduced pressure, and the residue subjected to column chromatography (silica gel, hexane/ethyl acetate, 2:1) to afford **15** (361 mg, 72%) as a colorless solid; R_f_ = 0.40 (silica gel_,_ hexanes/ethyl acetate, 2:1); m.p.: 209 °C (lit: [21] 290 °C); MS (APCI): *m*/*z* (%) = 418.3 ([M+H]^+^, 21%).

### 3.2. Crystallization and X-ray Data Collection

Crystal of hCA I was obtained using the hanging drop vapor diffusion method using a 24-well Linbro plate. Then, 2 µL of 10 mg/mL solution of hCA I in Tris-HCl 20 mM pH 9.0 was mixed with 2 µL of a solution of 28–31% PEG4000, 0.2 M sodium acetate, and 0.1 M Tris pH 8.5–9.0 and was equilibrated against the same solution at 296 K. The complex was prepared by soaking the hCA I native crystals in the mother liquor solution containing the inhibitor at a concentration of 10 mM for two days. All crystals were flash-frozen at 100 K using a solution obtained by adding 15% (*v*/*v*) glycerol to the mother liquor solution as cryoprotectant. Data on crystal of the complex were collected using synchrotron radiation at the XRD2 beamline at Elettra Synchrotron (Trieste, Italy) with a wavelength of 1.0 Å and a DECTRIS Pilatus 6M detector. Data were integrated and scaled using the XDS program [30]. Data processing statistics are shown in Supporting Information.

### 3.3. Structure Determination

The crystal structure of hCA I (PDB accession code: 1JV0) without solvent molecules and other heteroatoms was used to obtain initial phases using Refmac5 [31]. Then, 5% of the unique reflections were selected randomly and excluded from the refinement data set for the purpose of R_free_ calculations. The initial |Fo − Fc| difference electron density maps unambiguously showed the inhibitor molecules. Refinements proceeded using normal protocols of positional, isotropic atomic displacement parameters alternating with manual building of the models using COOT [32]. The quality of the final models was assessed with COOT and RAMPAGE [33]. Crystal parameters and refinement data are summarized in Supporting Information. Atomic coordinates were deposited in the Protein Data Bank (PDB accession code: 8CDX, 8CDZ). Graphical representations were generated with Chimera [34].

## 4. Conclusions

In conclusion, the synthesized ureidobenzenesulfonamides exhibited remarkably favorable K_i_ values within the enzyme assay. Notably, the ureidobenzenesulfonamides featuring biphenyl (**4**) and benzylphenyl (**2** and **3**) substitutions demonstrated exceptional selectivity toward tumor-associated hCA IX and hCA XII. Compound **3** showed a low K_i_ for the latter enzyme of 1 nM.

## Figures and Tables

**Table 1:** K_i_ values (in nM): Inhibition of human carbonic anhydrase I, II, IX, XII of compounds **1**–**15** with the standard inhibitor acetazolamide (**AAZ**) and **SLC-0111** (positive control) and calculated selectivity of hCA IX and hCA XII against hCA I and hCA II, respectively. Selectivity was calculated as the ratio of the respective K_i_ values.

Cmp	hCA I	hCA II	hCA IX	S(I/IX)	S(II/IX)	hCA XII	S(I/XII)	S(II/XII)
**1**	242.8	33.5	42.9	5.66	0.78	15.5	15.66	2.16
**2**	785.2	356.2	15.2	51.66	23.43	6.4	122.69	55.66
**3**	78.3	61.5	8.2	9.55	7.50	1	78.30	61.50
**4**	6650	5034	188.6	35.26	26.69	32.9	202.13	153.01
**5**	94.4	56.5	149.7	0.63	0.38	79.6	1.19	0.71
**6**	84.5	56.8	210.4	0.40	0.27	90.4	0.93	0.63
**7**	263.3	53.6	165.3	1.59	0.32	6.7	39.30	8.00
**8**	871.6	595.2	134.3	6.49	4.43	86.5	10.08	6.88
**9**	447.7	98.6	183.7	2.44	0.54	45.9	9.75	2.15
**10**	86.2	84.9	84.9	1.02	1.00	2.9	29.72	29.28
**11**	693.9	497.1	57.7	12.03	8.62	6.8	102.04	73.10
**12**	948.5	209.2	207.7	4.57	1.01	63.9	14.84	3.27
**13**	773.3	63.5	216.7	3.57	0.29	60.7	12.74	1.05
**14**	368.1	84.4	174.9	2.10	0.48	53.4	6.89	1.58
**15**	591.1	300.4	161.4	3.66	1.86	14.2	41.63	21.15
**SLC-0111**	5080	960	45.1	112.6	21.3	4.5	1128.9	213.3
**AAZ**	250	12.1	25.8	9.69	0.47	5.7	43.86	2.12

## Data Availability

The data presented in this study are available on request from the corresponding author.

## Supplementary Materials

The following supporting information can be downloaded at: https://www.mdpi.com/article/10.3390/molecules28237782/s1.
