# Supplementary material for: Ureidobenzenesulfonamides as Selective Carbonic Anhydrase I, IX, and XII Inhibitors
Source: Molecules. 2023 Nov 26;28(23):7782. doi: 10.3390/molecules28237782 (PMC10707797; doi:10.3390/molecules28237782)
Supplement: Supplementary file 1 [file molecules-28-07782-s001.zip › molecules-2647082-supplementary.pdf]

Supplementary Material

Ureidobenzenesulfonamides as Selective Carbonic Anhydrase I, IX, and XII Inhibitors

Toni C. Denner [1], Andrea Angeli [2], Marta Ferraroni [3], Claudiu T. Supuran [2] and René Csuk [1,*]

[1] Organic Chemistry, Martin-Luther University Halle-Wittenberg, Kurt-Mothes, Str. 2, D-06120 Halle (Saale), Germany; toni-christopher.denner@chemie.uni-halle.de
[2] Neurofarba Department, University of Florence, Section of Pharmaceutical Sciences, Via Ugo Schiff 6, Sesto Florentino, 50019 Florence, Italy; andrea.angeli@unifi.it (A.A.); claudiu.supuran@unifi.it (C.T.S.)
[3] Department of Chemistry "Ugo Schiff", University of Florence, Via della Lastruccia 3-13, Sesto Fiorentino, 50019 Florence, Italy; marta.ferraroni@unifi.it
* Correspondence: rene.csuk@chemie.uni-halle.de; Tel.: +49-345-55-25660

Summary of Data Collection and Atomic Model Refinement Statistics for hCAI

	**hCAI + 8**	**hCAI + 10**
PDB ID	8CDX	8CDZ
Wavelength (Å)	1.00	1.00
Space Group	P212121	P212121
Unit cell (a, b, c, α, β, γ) (Å,°)	62.27, 70.98, 121.58<br>90.00, 90.00, 90.00	62.24, 71.51, 121.76<br>90.00, 90.00, 90.00
Limiting resolution (Å)	46.85-1.33 (1.37-1.33)	46.99-1.44 (1.47-1.44)
Unique reflections	122663 (8895)	97034 (6949)
Rmerge (%)	21.6 (133.3)	5.6 (148.8)
Rmeas (%)	22.5 (141.7)	5.9 (155.3)
Redundancy	12.0 (8.6)	11.6 (12.1)
Completeness overall (%)	99.9 (98.7)	97.3 (95.8)
<I/σ(I)>	16.93 (2.34)	19.49 (2.15)
CC (1/2)	99.9 (83.9)	99.9 (82.8)
**Refinement statistics**		
Resolution range (Å)	61.30-1.33	61.30-1.44
Rfactor (%)	14.15	13.45
Rfree(%)	17.94	18.02
r.m.s.d. bonds(Å)	0.0171	0.0153
r.m.s.d. angles (°)	2.00368	1.9258
**Ramachandran statistics** (%)		
Most favored	97.3	97.5
additionally allowed	2.7	2.5
outlier regions	0.0	0.0

**Average B factor (Å^2)**		
All atoms	20.301	30.178
inhibitors	20.326	36.611
solvent	30.715	41.586

Additional spectroscopic data

4-[3-(Naphthalen-1-yl)ureido]benzenesulfonamide (**1**)

IR (ATR): *ν* = 1686*m*, 1491*m*, 1300*m*, 1245*m*, 1212*m*, 1147*s*, 794*m*, 769*m*, 614*s*, 542*s* cm^{-1}; ^{1}H NMR (400 MHz, DMSO-d_6): δ = 9.40 (*s*, 1H, 8-H), 8.85 (*s*, 1H, 6-H), 8.10 (*d*, *J* = 8.4 Hz, 1H, 10-H), 7.97 (*d*, *J* = 7.5 Hz, 1H, 14-H), 7.93 (*d*, *J* = 7.9 Hz, 1H, 17-H), 7.79 – 7.72 (*m*, 2H, 3-H), 7.69 – 7.62 (*m*, 2H, 4-H), 7.69 – 7.64 (*m*, 1H, 12-H), 7.60 – 7.55 (*m*, 1H, 16-H), 7.55 – 7.48 (*m*, 1H, 11-H), 7.47 (*m*, 1H, 15-H), 7.21 (*s*, 2H, 1-H) ppm; ^{13}C NMR (100 MHz, DMSO-d_6): δ = 153.2 (C-7), 143.3 (C-5), 137.7 (C-9), 134.3 (C-2), 134.2 (C-13), 128.9 (C-10), 127.3 (C-3), 126.6, (C-14), 126.4 (C-11), 126.3 (C-15), 126.3 (C-16), 123.9 (C-18), 121.8 (C-17), 118.5 (C-12), 117.9 (C-4) ppm.

4-[3-(4-Benzylphenyl)ureido]benzenesulfonamide (**2**)

IR (ATR): *ν* = 3262*w*, 2362*w*,1651*m*,1590*m*, 1538*s*, 1388*m*, 1329*s*,1303*m*, 1220*m*, 1151*s*, 1096*m*, 587*s*, 546*s* cm^{-1}; ^{1}H NMR (500 MHz, DMSO-d_6): δ = 8.98 (*s*, 1H, 6-H), 8.68 (*s*, 1H, 8-H), 7.73 – 7.66 (*m*, 2H, 3-H), 7.60 – 7.54 (*m*, 2H, 4-H), 7.38 – 7.33 (*m*, 2H, 10-H), 7.26 (*t*, *J* = 7.6 Hz, 2H, 16-H), 7.22 – 7.18 (*m*, 2H, 15-H), 7.18 – 7.15 (*m*, 1H, 19-H), 7.16 (*s*, 2H, 1-H), 7.14 – 7.11 (*m*, 2H, 11-H), 3.86 (*s*, 2H, 13-H) ppm; ^{13}C NMR (126 MHz, DMSO-d_6): δ = 152.7 (C-7), 143.3 (C-5), 142.0 (C-14), 137.7 (C-9), 137.2 (C-2), 135.6 (C-12), 129.5 (C-3), 129.1 (C-16), 128.8 (C-15), 127.25 (C-12), 126.3 (C-19), 119.1 (C-10), 117.8 (C-4), 40.9 (C-13) ppm.

4-[3-(2-Benzylphenyl)ureido]benzenesulfonamide (**3**)

IR (ATR): *ν* = 3378*m*, 3347*w*, 3249*w*, 1654*s*, 1592*m*, 1533*s*, 1486*s*, 1339*s*, 1226*m*, 1156*s*, 1096*w*, 838*w*, 742*m*, 656*m*, 576*m*, 538*s* cm^{-1}; ^{1}H NMR (500 MHz, DMSO-d_6): δ 9.29 = (*s*, 1H, 6-H), 8.14 (*s*, 1H, 8-H), 7.75 – 7.72 (*m*, 1H, 12-H), 7.72 – 7.68 (*m*, 2H, 3-H), 7.61 – 7.54 (*m*, 2H, 4-H), 7.30 – 7.24 (*m*, 2H, 18-H), 7.21 – 7.18 (*m*, 1H, 14-H), 7.21 – 7.14 (*m*, 2H, 17-H), 7.17 (*s*, 2H, 1-H), 7.17 – 7.15 (*m*, 1H, 13-H), 7.06 – 7.03 (*m*, 1H, 11-H), 7.03 – 6.99 (*m*, 1H, 19-H), 3.97 (*s*, 2H, 15-H) ppm; ^{13}C NMR (126 MHz, DMSO-d_6): δ = 153.1 (C-7), 143.4 (C-5), 140.3 (C-9), 137.2 (C-2), 136.9 (C-10), 132.7 (C-16), 129.2 (C-17), 128.9 (C-18), 127.3 (C-3), 127.0 (C-14), 126.5 (C-13), 124.3 (C-11), 123.6 (C-12), 117.8 (C-4), 36.7 (C-15) ppm.

4-{3-[4-Methoxy-(1,1'-biphenyl)-3-yl]ureido}benzenesulfonamide (**4**)

IR (ATR): *ν* = 3370*m*, 3227*w*, 1709*m*, 1597*m*, 1540*s*, 1317*s*, 1247*m*, 1222*m*, 1151*s*, 820*s*, 755*s*, 697*s*, 617*s*, 531*s* cm^{-1}; ^{1}H NMR (500 MHz, DMSO-d_6): δ = 9.71 (*s*, 1H, 6-H), 8.49 (*d*, *J* = 2.3 Hz, 1H, 14-H), 8.41 (*s*, 1H, 8-H), 7.76 – 7.69 (*m*, 2H, 3-H), 7.65 – 7.59 (*m*, 2H, 4-H), 7.59 – 7.54 (*m*, 2H, 17-H), 7.47 – 7.39 (*m*, 2H, 18-H), 7.34 – 7.28 (*m*, 1H, 19-H), 7.26 (*dd*, *J* = 8.4, 2.3 Hz, 1H, 12-H), 7.18 (*s*, 2H, 13-H), 7.11 (*d*, *J* = 8.5 Hz, 1H, 11-H), 3.92 (*s*, 3H, 15-H) ppm; ^{13}C NMR (126 MHz, DMSO-d_6): δ = 152.7 (C-7), 147.9 (C-10), 143.3 (C-5), 140.7 (C-16), 137.3 (C-2), 133.2 (C-13), 129.3 (C-18), 129.1 (C-9), 127.4 (C-3), 127.3 (C-19), 126.7 (C-12), 120.8 (C-9), 117.7 (C-4), 117.3 (C-14), 111.7 (C-11), 56.5 (C-15) ppm.

4-[3-(4-Methoxyphenyl)ureido]benzenesulfonamide (**5**)

IR (ATR): *ν* = 3258 *m*, 1649*s*, 1592*m*, 1560*s*, 1305*s*, 1251*s*, 1161*s*, 828*m*, 594*m*, 544*s* cm^{-1}; ^{1}H NMR (400 MHz, DMSO-d_6): δ = 8.94 (*s*, 1H, 6-H), 8.56 (*s*, 1H, 8-H), 7.74 – 7.66 (*m*, 2H, 3-H), 7.61 – 7.54 (*m*, 2H, 4-H), 7.39 – 7.28 (*m*, 2H, 10-H), 7.16 (*s*, 2H, 1-H), 6.91 – 6.80 (*m*, 2H, 11-H), 3.70 (*s*, 3H, 13-H) ppm; ^{13}C NMR (101 MHz, DMSO-d_6): δ = 155.2 (C-12), 152.9 (C-7), 143.5 (C-5), 137.1 (C-2), 132.7 (C-9), 127.2 (C-3), 120.8 (C-10), 117.7 (C-4), 114.5 (C-11), 55.6 (C-13) ppm.

4-[3-(3-Methoxyphenyl)ureido]benzenesulfonamide (**6**)

IR (ATR): *ν* = 3392*w*, 3280 *w*, 1694*m*, 15901*m*, 1528*m*, 1286*m*, 1152*s*, 771*m*, 549scm^{-1}; ^{1}H NMR (500 MHz, DMSO-d_6): δ = 9.01 (*s*, 1H, 6-H), 8.76 (*s*, 1H, 8-H), 7.74 – 7.69 (*m*, 2H, 3-H), 7.62 – 7.55 (*m*, 2H, 4-H), 7.20 – 7.17 (*m*, 1H, 10-H), 7.18 (*s*, 2H, 1-H), 7.18 – 7.15 (*m*, 1H, 13-H), 6.93 (*ddd*, *J* = 8.1, 2.0, 0.9 Hz, 1H, 14-H), 6.56 (*ddd*, *J* = 8.2, 2.5, 0.9 Hz, 1H, 12-H), 3.72 (*s*, 3H, 15-H) ppm; ^{13}C NMR (126 MHz, DMSO-d_6): δ = 160.2 (C-11), 152.6 (C-7), 143.2 (C-5), 141.0 (C-9), 137.3 (C-2), 130.1 (C-13), 127.3 (C-3), 117.9 (C-4), 111.2 (C-14), 108.1 (C-12), 104.6 (C-10), 55.4 (C-15) ppm.

4-[3-(3-Fluorophenyl)ureido]benzenesulfonamide (**7**)

IR (ATR): *ν* = 3297*w*, 1701*m*, 1541*m*, 1312*m*, 1157*s*, 829 *m*, 773*m*, 657*m*, 540*m*, 526*s* cm^{-1}; ^{1}H NMR (500 MHz, DMSO-d_6): δ = 9.09 (*s*, 1H, 6-H), 8.99 (*s*, 1H, 8-H), 7.76 – 7.69 (*m*, 2H, 3-H), 7.63 – 7.56 (*m*, 2H, 4-H), 7.47 (*dt*, *J* = 11.9, 2.3 Hz, 1H, 10-H), 7.30 (*dt, J* = 8.2, 6.9 Hz, 1H, 12-H), 7.19 (*s*, 2H, 1-H), 7.15 – 7.10 (*m*, 1H, 14-H), 6.83 – 6.75 (*m*, 1H, 13-H) ppm; ^{13}C NMR (126 MHz, DMSO-d_6): δ = 162.8 (*d*, *J* = 240.8 Hz, C-11), 152.6 (C-7), 143.01 (C-5), 141.7 (*d*, *J* = 11.1 Hz, C-9), 137.6, 130.8 (*d*, *J* = 9.6 Hz, C-13), 127.3 (C-3), 118.1 (C-4), 114.6 (*d*, *J* = 2.7 Hz, C-14), 109.0 (*d*, *J* = 21.1 Hz, C-10), 105.6 (*d*, *J* = 26.4 Hz, C-12) ppm.

4-(3-Ethylureido)benzenesulfonamide (**8**)

IR (ATR): *v* = 3295*w*, 1661*m*, 1593*m*, 1535*m*, 1317*m*, 1233*m*, 1159*s*, 1097*m*, 832*m*, 741*m*, 589*m*, 537*s* cm^{-1}; ^{1}H NMR (400 MHz, DMSO-d_6): δ = 8.79 (*s*, 1H, 6-H), 7.68 – 7.60 (*m*, 2H, 3-H), 7.55 – 7.47 (*m*, 2H, 4-H), 7.11 (*s*, 2H, 1-H), 6.22 (*t*, *J* = 5.6 Hz, 1H, 8-H), 3.10 (*qd*, *J* = 7.1, 5.5 Hz, 2H, 9-H), 1.04 (*t*, *J* = 7.2 Hz, 3H, 10-H) ppm; ^{13}C NMR (101 MHz, DMSO-d_6): δ = 155.16 (C-7), 144.18 (C-5), 136.36 (C-2), 127.16 (C-3), 117.20 (C-4), 34.44 (C-9), 15.77 (C-10) ppm.

4-[3-Isopropylureido]benzenesulfonamide (**9**)

IR (ATR): *v* = 3325*w*, 1634*s*, 1133*m*, 1247*m*, 1149*s*, 1099*m*, 739*m*, 587*m*, 539*m* cm^{-1}; ^{1}H NMR (400 MHz, DMSO-d_6): δ = 8.65 (*s*, 1H, 6-H), 7.68 – 7.60 (*m*, 2H, 3-H), 7.53 – 7.46 (*m*, 2H, 4-H), 7.11 (*s*, 2H, 1-H), 6.12 (*d*, *J* = 7.5 Hz, 1H, 8-H), 3.82 – 3.66 (*m*, 1H, 9-H), 1.08 (*d*, *J* = 6.6 Hz, 5H, 10-H) ppm; ^{13}C NMR (101 MHz, DMSO-d_6): δ = 154.5 (C-7), 144.1 (C-5), 136.4 (C-2), 127.2 (C-3), 117.1 (C-4), 41.5 (C-9), 23.3 (C-10) ppm.

4-(3-Butylureido)benzenesulfonamide (**10**)

IR (ATR): *v* = 3403*w*, 3296*m*, 3088*w*, 2963*w*, 1687*s*, 1593*m*, 1533*m*, 1324*m*, 1228*m*, 1154*s*, 830*s*, 598*m*, 542*s* cm^{-1}; ^{1}H NMR (500 MHz, DMSO-d_6): δ = 8.76 (*s*, 1H, 6-H), 7.67 – 7.61 (*m*, 2H, 3-H), 7.54 – 7.47 (*m*, 2H, 4-H), 7.11 (*s*, 2H, 1-H), 6.23 (*t*, *J* = 5.6 Hz, 1H, 8-H), 3.07 (*q*, *J* = 6.9 Hz, 2H, 9-H), 1.45 – 1.35 (*m*, 2H, 10-H), 1.35 – 1.24 (*m*, 2H, 11-H), 0.88 (*t*, *J* = 7.3 Hz, 3H, 12-H) ppm; ^{13}C NMR (126 MHz, DMSO-d_6): δ = 155.2 (C-7), 144.2 (C-5), 136.4 (C-2), 127.2 (C-3), 117.2 (C-4), 39.2 (C-9), 32.2 (C-10), 20.0 (C-11), 14.1 (C-12) ppm.

4-(3-Hexylureido)benzenesulfonamide (**11**)

IR (ATR): *v* = 3410*m*, 3352*m*, 3201*w*, 2938*m*, 2850*m*, 1682*s*, 1596*m*, 1307*s*, 1237*m*, 1151*s*, 825*m*, 590*m*, 541*m*, 513*m* cm^{-1}; ^{1}H NMR (400 MHz, DMSO-d_6): δ = 8.77 (*s*, 1H, 6-H), 7.68 – 7.60 (*m*, 2H, 3-H), 7.54 – 7.46 (*m*, 2H, 4-H), 7.12 (*s*, 2H, 1-H), 6.24 (*t*, *J* = 5.7 Hz, 1H, 8-H), 3.06 (*dt, J* = 6.9, 5.7 Hz, 2H, 9-H), 1.40 (*tt, J* = 7.2, 3.8 Hz, 2H, 10-H), 1.30 – 1.21 (*m*, 4H, 12-H+13-H), 1.26 – 1.25 (*m*, 2H, 13-H), 0.89 – 0.81 (*m*, 3H, 14-H) ppm; ^{13}C NMR (101 MHz, DMSO-d_6): δ = 155.2 (C-7), 144.2 (C-5), 136.3 (C-2), 127.2 (C-3), 117.2 (C-4), 39.5 (C-9), 31.4 (C-12), 30.0 (C-10), 26.5 (C-11), 22.5 (C-13), 14.4 (C-14) ppm.

N-[(4-Sulfamoylphenyl)carbamoyl]benzamide (**12**)

IR (ATR): *v* = 3339*w*, 3245*w*, 1700*s*, 1593*m*, 1160*m*, 1097*m*, 750*m*, 704*s*, 544*s* cm^{-1}; ^{1}H NMR (500 MHz, DMSO-d_6): δ = 11.12 (*s*, 1H, 8-H), 11.03 (*s*, 1H, 6-H), 8.04 – 7.98 (*m*, 2H, 11-H),

7.82 – 7.73 (*m*, 4H, 3-H+4-H), 7.69 – 7.62 (*m*, 1H), 7.57 – 7.50 (*m*, 2H, 4-H), 7.27 (*s*, 2H, 1-H) ppm; ^{13}C NMR (126 MHz, DMSO-d$_6$): δ = 169.2 (C-9), 151.6 (C-7), 141.1 (C-4), 139.3 (C-2), 133.6 (C-10), 132.6 (C-13), 129.1 (C-4), 128.8 (C-12), 127.3 (C-11), 119.9 (C-4) ppm.

Ethyl 4-[3-(4-sulfamoylphenyl)ureido]benzoate (**13**)

IR (ATR): *ν* = 3298*w*, 1698*s*, 1591*s*, 1411*m*, 1370*m*, 1150*m*, 1100*m*, 1016*m*, 902*w*, 768*m*, 642*m*, 572*m*, 538*s* cm^{-1}; ^{1}H NMR (500 MHz, DMSO-d$_6$): δ = 9.18 (*s*, 1H, 8-H), 9.14 (*s*, 1H, 6-H), 7.91 – 7.86 (*m*, 2H, 11-H), 7.77 – 7.70 (*m*, 2H, 3-H), 7.64 – 7.60 (*m*, 2H, 4-H), 7.60 – 7.56 (*m*, 2H, 10-H), 7.19 (*s*, 2H, 1-H), 4.26 (*q*, *J* = 7.0 Hz, 2H, 14-H), 1.29 (*t*, *J* = 7.1 Hz, 3H, 15-H) ppm; ^{13}C NMR (126 MHz, DMSO-d$_6$): δ = 165.8 (C-13), 152.4 (C-7), 144.4 (C-9), 142.9 (C-5), 137.7 (C-2), 130.8 (C-11), 127.3 (C-3), 123.56 (C-12), 118.2 (C-4), 118.0 (C-10), 60.8 (C-14), 14.7 (C-15) ppm.

4-[3-(4-Sulfamoylphenyl)ureido]benzoic acid (**14**)

IR (ATR): *ν* = 1655*s*, 1590*s*, 1535*s*, 1329*m*, 1218*m*, 1151*s*, 764*s*, 640*m*, 541*s* cm^{-1}; ^{1}H NMR (500 MHz, DMSO-d$_6$): δ = 12.60 (*s*, 1H, 14-H), 9.15 (*s*, 1H, 8-H), 9.15 (*s*, 1H, 6-H), 7.92 – 7.84 (*m*, 2H, 11-H), 7.77 – 7.69 (*m*, 2H, 3-H), 7.64 – 7.59 (*m*, 2H, 4-H), 7.58 – 7.54 (*m*, 2H, 10-H), 7.19 (*s*, 2H, 1-H) ppm; ^{13}C NMR (126 MHz, DMSO-d$_6$): δ = 167.4 (C-13), 152.5 (C-7), 144.1 (C-9), 142.93 (C-5), 137.65 (C-2), 131.0 (C-10), 127.3 (C-3), 124.5 (C-12), 118.2 (C-4), 117.9 (C-11) ppm.

4-[3-(5-Chloro-2-phenoxyphenyl)ureido]benzenesulfonamide (**15**)

IR (ATR): *ν* = 3361*m*, 1713*m*, 1590*s*, 1529*s*, 1473*m*, 1310*m*, 1218*s*, 1146*m*, 838*m*, 612*m*, 535*s*, 492*s* cm^{-1}; ^{1}H NMR (500 MHz, DMSO-d$_6$): δ = 9.68 (*s*, 1H, 6-H), 8.74 (*s*, 1H, 8-H), 8.36 (*d*, *J* = 2.6 Hz, 1H, 10-H), 7.75 – 7.69 (*m*, 2H, 3-H), 7.61 – 7.54 (*m*, 2H, 4-H), 7.46 – 7.38 (*m*, 2H, 17-H), 7.19 (*s*, 2H, 1-H), 7.20 – 7.16 (*m*, 1H, 18-H), 7.11 – 7.04 (*m*, 2H, 16-H), 7.00 (*dd*, *J* = 8.7, 2.6 Hz, 1H, 12-H), 6.82 (*d*, *J* = 8.7 Hz, 1H, 13-H) ppm; ^{13}C NMR (126 MHz, DMSO-d$_6$) δ 156.7 (C-15), 152.4 (C-7), 144.5 (C-14), 142.8 (C-5), 137.7 (C-2), 132.6 (C-28), 130.7 (C-17), 128.0 (C-11), 127.4 (C-3), 124.6 (C-15), 122.4 (C-12), 119.8 (C-13), 119.2 (C-10), 119.1 (C-16), 118.0 (C-4) ppm.

NMR spectra and calculated ADME parameters

4-[3-(Naphthalen-1-yl)ureido]benzenesulfonamide (**1**)

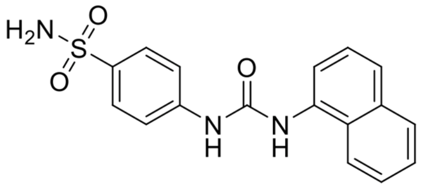

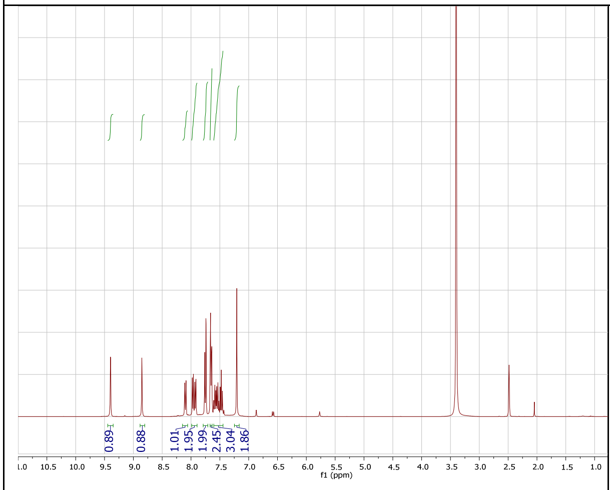

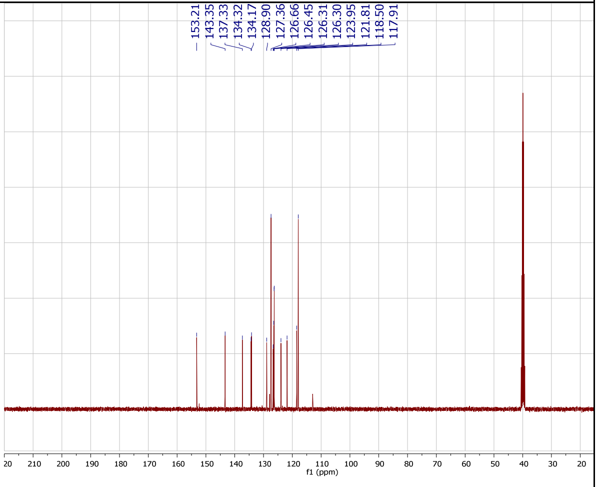

Molecule 1

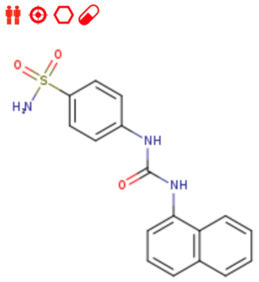

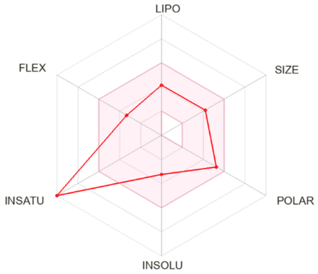

SMILES O=C(Nc1cccc2c1cccc2)Nc1ccc(cc1)S(=O)(=O)N

Physicochemical Properties	
Formula	C17H15N3O3S
Molecular weight	341.38 g/mol
Num. heavy atoms	24
Num. arom. heavy atoms	16
Fraction Csp3	0.00
Num. rotatable bonds	5
Num. H-bond acceptors	4
Num. H-bond donors	3
Molar Refractivity	93.66
TPSA	109.67 Å²

Lipophilicity	
Log $P_{o/w}$ (iLOGP)	1.67
Log $P_{o/w}$ (XLOGP3)	1.77
Log $P_{o/w}$ (WLOGP)	3.83
Log $P_{o/w}$ (MLOGP)	2.21
Log $P_{o/w}$ (SILICOS-IT)	0.95
Consensus Log $P_{o/w}$	2.08

Water Solubility	
Log *S* (ESOL)	-3.24
Solubility	1.99e-01 mg/ml ; 5.82e-04 mol/l
Class	Soluble
Log *S* (Ali)	-3.69
Solubility	6.95e-02 mg/ml ; 2.04e-04 mol/l
Class	Soluble
Log *S* (SILICOS-IT)	-6.17
Solubility	2.28e-04 mg/ml ; 6.68e-07 mol/l
Class	Poorly soluble

Pharmacokinetics	
GI absorption	High
BBB permeant	No
P-gp substrate	No
CYP1A2 inhibitor	No
CYP2C19 inhibitor	No
CYP2C9 inhibitor	Yes
CYP2D6 inhibitor	No
CYP3A4 inhibitor	No
Log K_p (skin permeation)	-7.13 cm/s

Druglikeness	
Lipinski	Yes; 0 violation
Ghose	Yes
Veber	Yes
Egan	Yes
Muegge	Yes
Bioavailability Score	0.55

Medicinal Chemistry	
PAINS	0 alert
Brenk	0 alert
Leadlikeness	Yes
Synthetic accessibility	2.41

4-[3-(4-Benzylphenyl)ureido]benzenesulfonamide (**2**)

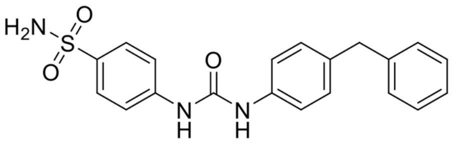

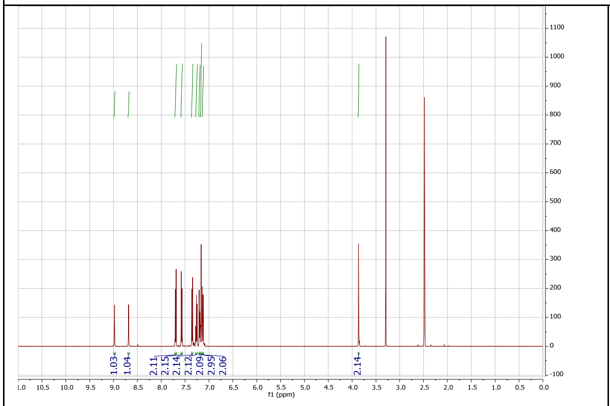

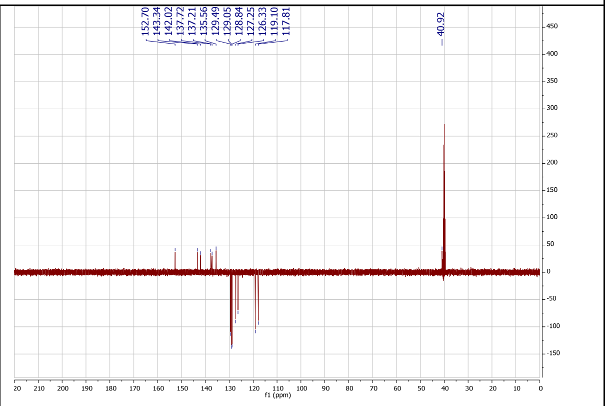

Molecule 2

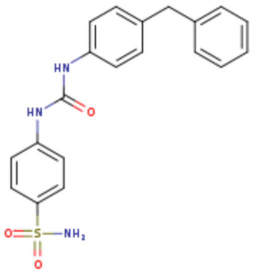

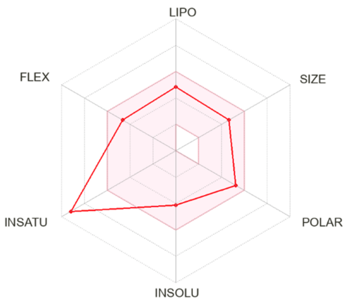

SMILES O=C(Nc1ccc(cc1)S(=O)(=O)N)Nc1ccc(cc1)Cc1ccccc1

Physicochemical Properties	
Formula	C20H19N3O3S
Molecular weight	381.45 g/mol
Num. heavy atoms	27
Num. arom. heavy atoms	18
Fraction Csp3	0.05
Num. rotatable bonds	7
Num. H-bond acceptors	4
Num. H-bond donors	3
Molar Refractivity	105.61
TPSA	109.67 Å²

Lipophilicity	
Log $P_{o/w}$ (iLOGP)	2.05
Log $P_{o/w}$ (XLOGP3)	2.97
Log $P_{o/w}$ (WLOGP)	4.27
Log $P_{o/w}$ (MLOGP)	2.87
Log $P_{o/w}$ (SILICOS-IT)	1.89
Consensus Log $P_{o/w}$	2.81

Water Solubility	
Log *S* (ESOL)	-4.11
Solubility	2.98e-02 mg/ml ; 7.81e-05 mol/l
Class	Moderately soluble
Log *S* (Ali)	-4.94
Solubility	4.42e-03 mg/ml ; 1.16e-05 mol/l
Class	Moderately soluble
Log *S* (SILICOS-IT)	-7.39
Solubility	1.55e-05 mg/ml ; 4.06e-08 mol/l
Class	Poorly soluble

Pharmacokinetics	
GI absorption	High
BBB permeant	No
P-gp substrate	No
CYP1A2 inhibitor	No
CYP2C19 inhibitor	Yes
CYP2C9 inhibitor	Yes
CYP2D6 inhibitor	No
CYP3A4 inhibitor	No
Log K_p (skin permeation)	-6.52 cm/s

Druglikeness	
Lipinski	Yes; 0 violation
Ghose	Yes
Veber	Yes
Egan	Yes
Muegge	Yes
Bioavailability Score	0.55

Medicinal Chemistry	
PAINS	0 alert
Brenk	0 alert
Leadlikeness	No; 1 violation: MW>350
Synthetic accessibility	2.61

4-[3-(2-Benzylphenyl)ureido]benzenesulfonamide (**3**)

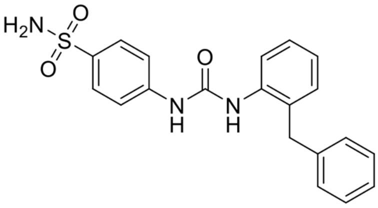

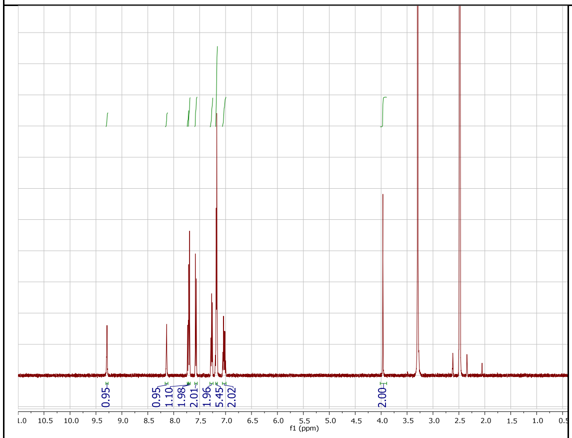

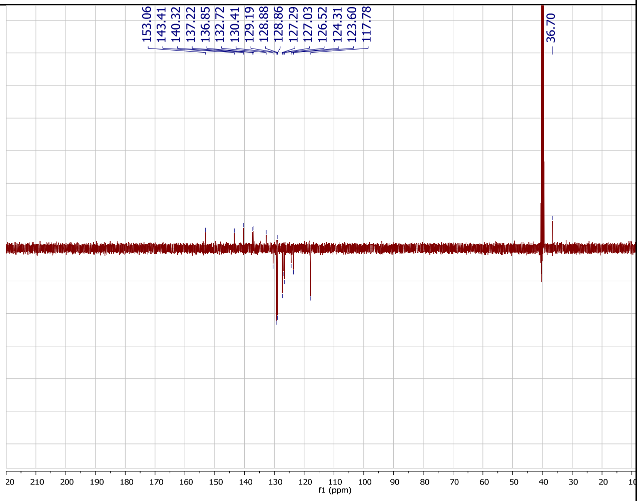

Molecule 5

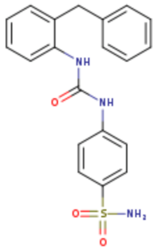

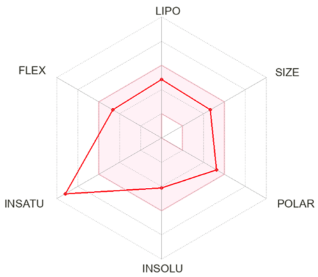

SMILES O=C(Nc1ccccc1Cc1ccccc1)Nc1ccc(cc1)S(=O)(=O)N

Physicochemical Properties	
Formula	C20H19N3O3S
Molecular weight	381.45 g/mol
Num. heavy atoms	27
Num. arom. heavy atoms	18
Fraction Csp3	0.05
Num. rotatable bonds	7
Num. H-bond acceptors	4
Num. H-bond donors	3
Molar Refractivity	105.61
TPSA	109.67 Å²
**Lipophilicity**	
Log $P_{o/w}$ (iLOGP)	1.60
Log $P_{o/w}$ (XLOGP3)	2.97
Log $P_{o/w}$ (WLOGP)	4.27
Log $P_{o/w}$ (MLOGP)	2.87
Log $P_{o/w}$ (SILICOS-IT)	1.89
Consensus Log $P_{o/w}$	2.72

Water Solubility	
Log *S* (ESOL)	-4.11
Solubility	2.98e-02 mg/ml ; 7.81e-05 mol/l
Class	Moderately soluble
Log *S* (Ali)	-4.94
Solubility	4.42e-03 mg/ml ; 1.16e-05 mol/l
Class	Moderately soluble
Log *S* (SILICOS-IT)	-7.39
Solubility	1.55e-05 mg/ml ; 4.06e-08 mol/l
Class	Poorly soluble
**Pharmacokinetics**	
GI absorption	High
BBB permeant	No
P-gp substrate	No
CYP1A2 inhibitor	No
CYP2C19 inhibitor	Yes
CYP2C9 inhibitor	Yes
CYP2D6 inhibitor	No
CYP3A4 inhibitor	No
Log K_p (skin permeation)	-6.52 cm/s
**Druglikeness**	
Lipinski	Yes; 0 violation
Ghose	Yes
Veber	Yes
Egan	Yes
Muegge	Yes
Bioavailability Score	0.55
**Medicinal Chemistry**	
PAINS	0 alert
Brenk	0 alert
Leadlikeness	No; 1 violation: MW>350
Synthetic accessibility	2.77

4-{3-[4-Methoxy-(1,1'-biphenyl)-3-yl]ureido}benzenesulfonamide (**4**)

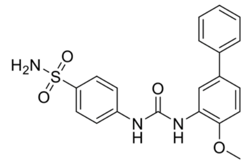

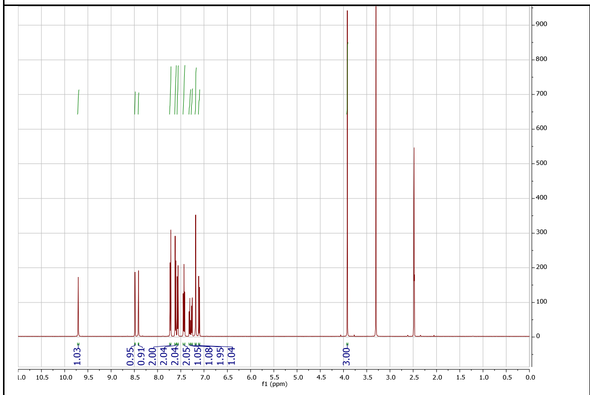

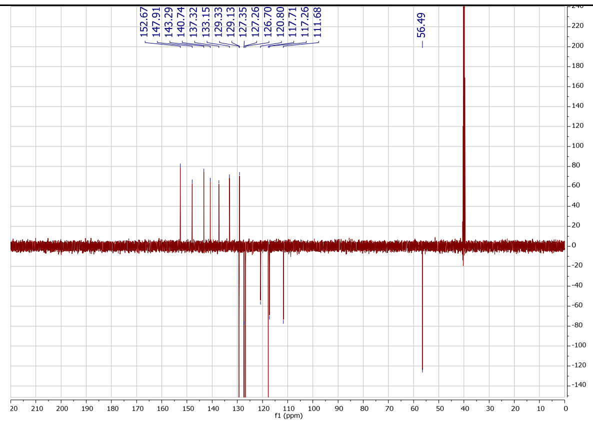

Molecule 3

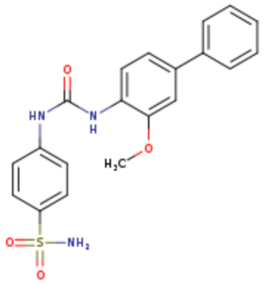

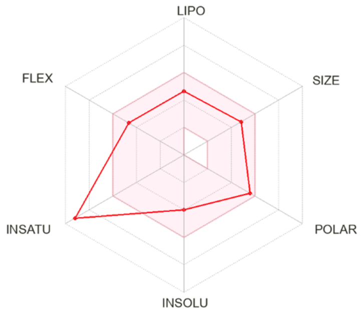

SMILES COc1cc(ccc1NC(=O)Nc1ccc(cc1)S(=O)(=O)N)c1ccccc1

Physicochemical Properties	
Formula	C20H19N3O4S
Molecular weight	397.45 g/mol
Num. heavy atoms	28
Num. arom. heavy atoms	18
Fraction Csp3	0.05
Num. rotatable bonds	7
Num. H-bond acceptors	5
Num. H-bond donors	3
Molar Refractivity	108.09
TPSA	118.90 Å²

Lipophilicity	
Log $P_{o/w}$ (iLOGP)	2.52
Log $P_{o/w}$ (XLOGP3)	2.65
Log $P_{o/w}$ (WLOGP)	4.35
Log $P_{o/w}$ (MLOGP)	2.34
Log $P_{o/w}$ (SILICOS-IT)	1.56
Consensus Log $P_{o/w}$	2.68

Water Solubility	
Log *S* (ESOL)	-3.99
Solubility	4.09e-02 mg/ml ; 1.03e-04 mol/l
Class	Soluble
Log *S* (Ali)	-4.80
Solubility	6.33e-03 mg/ml ; 1.59e-05 mol/l
Class	Moderately soluble
Log *S* (SILICOS-IT)	-7.10
Solubility	3.14e-05 mg/ml ; 7.91e-08 mol/l
Class	Poorly soluble

Pharmacokinetics	
GI absorption	High
BBB permeant	No
P-gp substrate	No
CYP1A2 inhibitor	No
CYP2C19 inhibitor	No
CYP2C9 inhibitor	Yes
CYP2D6 inhibitor	No
CYP3A4 inhibitor	No
Log K_p (skin permeation)	-6.84 cm/s

Druglikeness	
Lipinski	Yes; 0 violation
Ghose	Yes
Veber	Yes
Egan	Yes
Muegge	Yes
Bioavailability Score	0.55

Medicinal Chemistry	
PAINS	0 alert
Brenk	0 alert
Leadlikeness	No; 1 violation: MW>350
Synthetic accessibility	2.79

4-[3-(4-Methoxyphenyl)ureido]benzenesulfonamide (**5**)

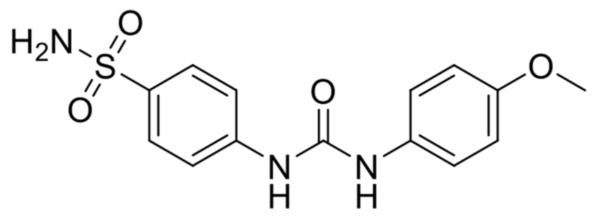

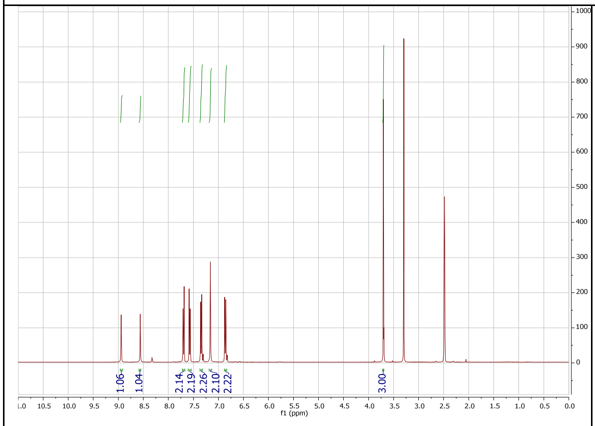

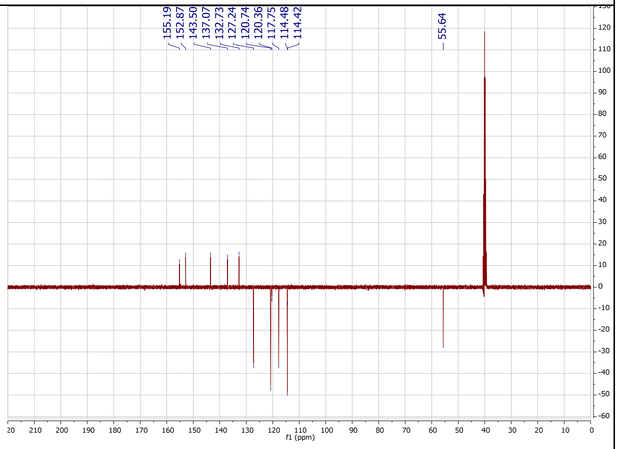

Molecule 5

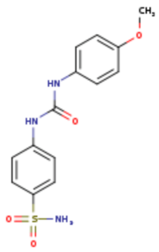

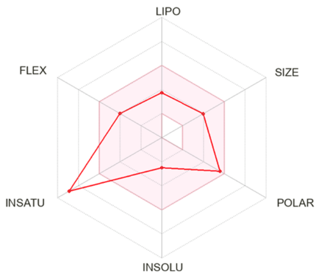

SMILES COc1ccc(cc1)NC(=O)Nc1ccc(cc1)S(=O)(=O)N

Physicochemical Properties	
Formula	C14H15N3O4S
Molecular weight	321.35 g/mol
Num. heavy atoms	22
Num. arom. heavy atoms	12
Fraction Csp3	0.07
Num. rotatable bonds	6
Num. H-bond acceptors	5
Num. H-bond donors	3
Molar Refractivity	82.65
TPSA	118.90 Å²

Lipophilicity	
Log $P_{o/w}$ (iLOGP)	1.60
Log $P_{o/w}$ (XLOGP3)	1.03
Log $P_{o/w}$ (WLOGP)	2.69
Log $P_{o/w}$ (MLOGP)	1.11
Log $P_{o/w}$ (SILICOS-IT)	-0.04
Consensus Log $P_{o/w}$	1.28

Water Solubility	
Log *S* (ESOL)	-2.49
Solubility	1.04e+00 mg/ml ; 3.24e-03 mol/l
Class	Soluble
Log *S* (Ali)	-3.12
Solubility	2.45e-01 mg/ml ; 7.64e-04 mol/l
Class	Soluble
Log *S* (SILICOS-IT)	-4.64
Solubility	7.38e-03 mg/ml ; 2.30e-05 mol/l
Class	Moderately soluble

Pharmacokinetics	
GI absorption	High
BBB permeant	No
P-gp substrate	No
CYP1A2 inhibitor	No
CYP2C19 inhibitor	No
CYP2C9 inhibitor	Yes
CYP2D6 inhibitor	No
CYP3A4 inhibitor	No
Log K_p (skin permeation)	-7.53 cm/s

Druglikeness	
Lipinski	Yes; 0 violation
Ghose	Yes
Veber	Yes
Egan	Yes
Muegge	Yes
Bioavailability Score	0.55

Medicinal Chemistry	
PAINS	0 alert
Brenk	0 alert
Leadlikeness	Yes
Synthetic accessibility	2.24

4-[3-(3-Methoxyphenyl)ureido]benzenesulfonamide (**6**)

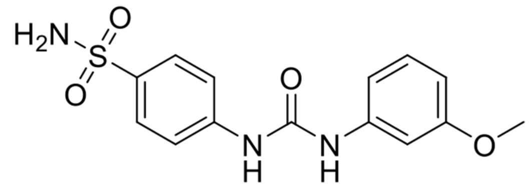

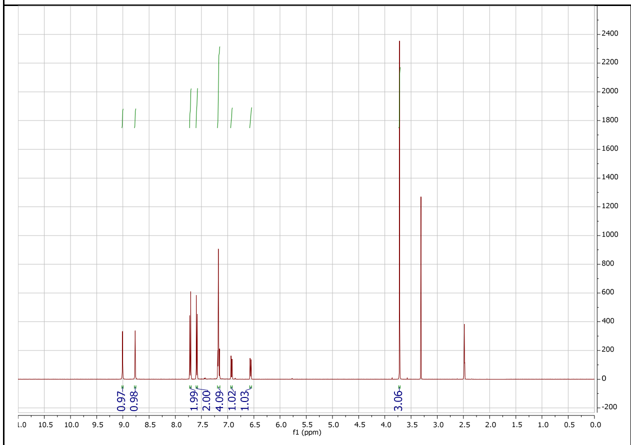

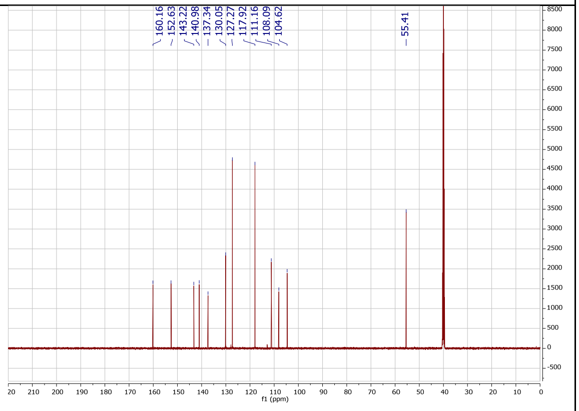

Molecule 1

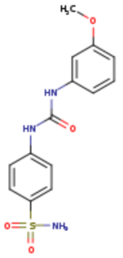

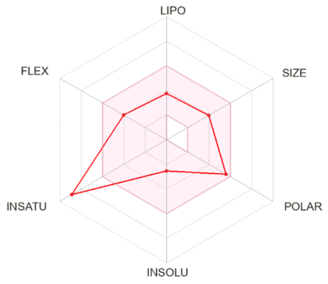

SMILES COc1cccc(c1)NC(=O)Nc1ccc(cc1)S(=O)(=O)N

Physicochemical Properties	
Formula	C14H15N3O4S
Molecular weight	321.35 g/mol
Num. heavy atoms	22
Num. arom. heavy atoms	12
Fraction Csp3	0.07
Num. rotatable bonds	6
Num. H-bond acceptors	5
Num. H-bond donors	3
Molar Refractivity	82.65
TPSA	118.90 Å²

Lipophilicity	
Log $P_{o/w}$ (iLOGP)	1.59
Log $P_{o/w}$ (XLOGP3)	1.09
Log $P_{o/w}$ (WLOGP)	2.69
Log $P_{o/w}$ (MLOGP)	1.11
Log $P_{o/w}$ (SILICOS-IT)	-0.04
Consensus Log $P_{o/w}$	1.29

Water Solubility	
Log *S* (ESOL)	-2.53
Solubility	9.56e-01 mg/ml ; 2.97e-03 mol/l
Class	Soluble
Log *S* (Ali)	-3.18
Solubility	2.13e-01 mg/ml ; 6.62e-04 mol/l
Class	Soluble
Log *S* (SILICOS-IT)	-4.64
Solubility	7.38e-03 mg/ml ; 2.30e-05 mol/l
Class	Moderately soluble

Pharmacokinetics	
GI absorption	High
BBB permeant	No
P-gp substrate	No
CYP1A2 inhibitor	No
CYP2C19 inhibitor	No
CYP2C9 inhibitor	Yes
CYP2D6 inhibitor	No
CYP3A4 inhibitor	No
Log K_p (skin permeation)	-7.49 cm/s

Druglikeness	
Lipinski	Yes; 0 violation
Ghose	Yes
Veber	Yes
Egan	Yes
Muegge	Yes
Bioavailability Score	0.55

Medicinal Chemistry	
PAINS	0 alert
Brenk	0 alert
Leadlikeness	Yes
Synthetic accessibility	2.42

4-[3-(3-Fluorophenyl)ureido]benzenesulfonamide (**7**)

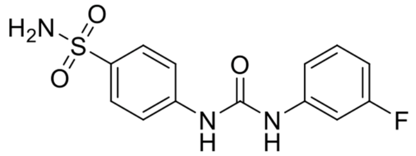

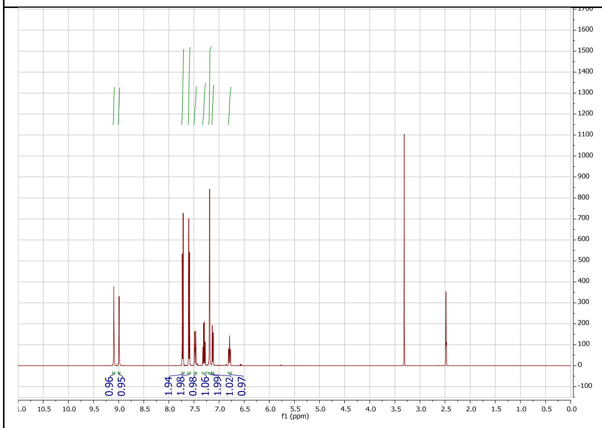

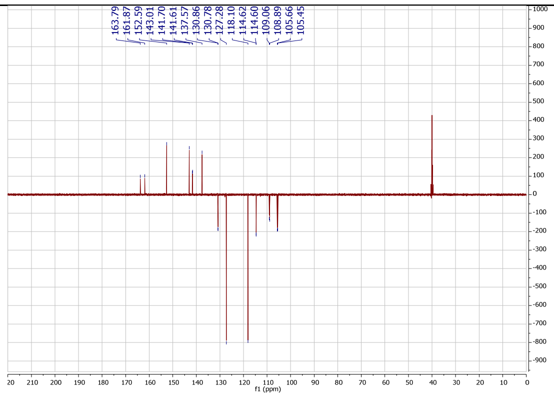

Molecule 2

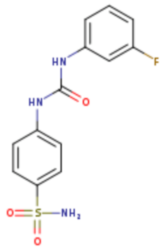

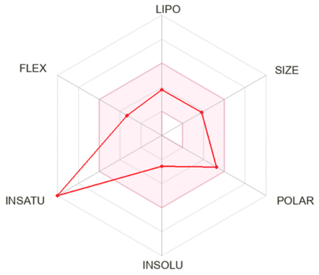

SMILES O=C(Nc1cccc(c1)F)Nc1ccc(cc1)S(=O)(=O)N

Physicochemical Properties	
Formula	C13H12FN3O3S
Molecular weight	309.32 g/mol
Num. heavy atoms	21
Num. arom. heavy atoms	12
Fraction Csp3	0.00
Num. rotatable bonds	5
Num. H-bond acceptors	5
Num. H-bond donors	3
Molar Refractivity	76.12
TPSA	109.67 Å²

Lipophilicity	
Log $P_{o/w}$ (iLOGP)	1.24
Log $P_{o/w}$ (XLOGP3)	1.15
Log $P_{o/w}$ (WLOGP)	3.24
Log $P_{o/w}$ (MLOGP)	1.78
Log $P_{o/w}$ (SILICOS-IT)	0.34
Consensus Log $P_{o/w}$	1.55

Water Solubility	
Log *S* (ESOL)	-2.58
Solubility	8.23e-01 mg/ml ; 2.66e-03 mol/l
Class	Soluble
Log *S* (Ali)	-3.05
Solubility	2.77e-01 mg/ml ; 8.96e-04 mol/l
Class	Soluble
Log *S* (SILICOS-IT)	-4.80
Solubility	4.94e-03 mg/ml ; 1.60e-05 mol/l
Class	Moderately soluble

Pharmacokinetics	
GI absorption	High
BBB permeant	No
P-gp substrate	No
CYP1A2 inhibitor	No
CYP2C19 inhibitor	No
CYP2C9 inhibitor	No
CYP2D6 inhibitor	No
CYP3A4 inhibitor	No
Log K_p (skin permeation)	-7.37 cm/s

Druglikeness	
Lipinski	Yes; 0 violation
Ghose	Yes
Veber	Yes
Egan	Yes
Muegge	Yes
Bioavailability Score	0.55

Medicinal Chemistry	
PAINS	0 alert
Brenk	0 alert
Leadlikeness	Yes
Synthetic accessibility	2.31

4-(3-Ethylureido)benzenesulfonamide (**8**)

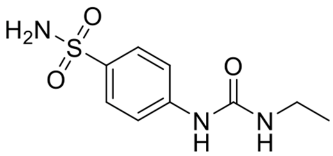

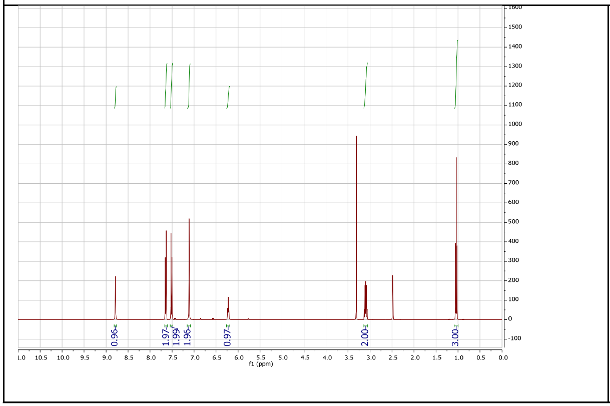

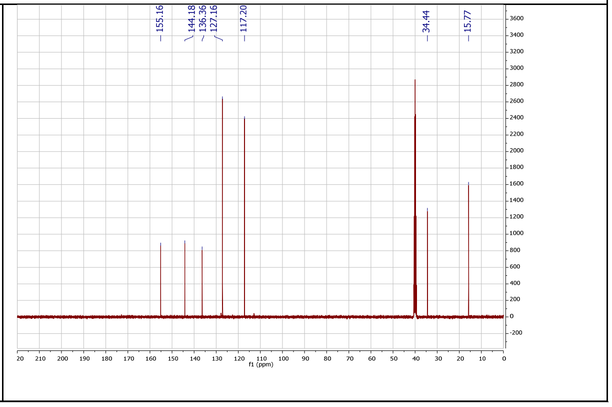

Molecule 2

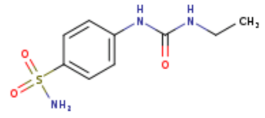

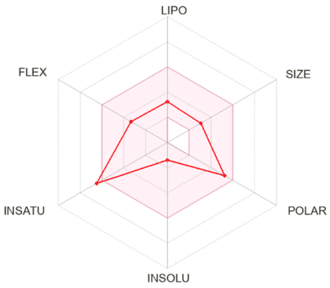

SMILES CCNC(=O)Nc1ccc(cc1)S(=O)(=O)N

Physicochemical Properties	
Formula	C9H13N3O3S
Molecular weight	243.28 g/mol
Num. heavy atoms	16
Num. arom. heavy atoms	6
Fraction Csp3	0.22
Num. rotatable bonds	5
Num. H-bond acceptors	4
Num. H-bond donors	3
Molar Refractivity	59.75
TPSA	109.67 Å²

Lipophilicity	
Log $P_{o/w}$ (iLOGP)	0.90
Log $P_{o/w}$ (XLOGP3)	0.16
Log $P_{o/w}$ (WLOGP)	1.37
Log $P_{o/w}$ (MLOGP)	0.19
Log $P_{o/w}$ (SILICOS-IT)	-0.86
Consensus Log $P_{o/w}$	0.35

Water Solubility	
Log *S* (ESOL)	-1.40
Solubility	9.76e+00 mg/ml ; 4.01e-02 mol/l
Class	Very soluble
Log *S* (Ali)	-2.02
Solubility	2.32e+00 mg/ml ; 9.54e-03 mol/l
Class	Soluble
Log *S* (SILICOS-IT)	-2.81
Solubility	3.73e-01 mg/ml ; 1.53e-03 mol/l
Class	Soluble

Pharmacokinetics	
GI absorption	High
BBB permeant	No
P-gp substrate	No
CYP1A2 inhibitor	No
CYP2C19 inhibitor	No
CYP2C9 inhibitor	No
CYP2D6 inhibitor	No
CYP3A4 inhibitor	No
Log K_p (skin permeation)	-7.67 cm/s

Druglikeness	
Lipinski	Yes; 0 violation
Ghose	Yes
Veber	Yes
Egan	Yes
Muegge	Yes
Bioavailability Score	0.55

Medicinal Chemistry	
PAINS	0 alert
Brenk	0 alert
Leadlikeness	No; 1 violation: MW<250
Synthetic accessibility	1.99

4-(3-Isopropylureido)benzenesulfonamide (**9**)

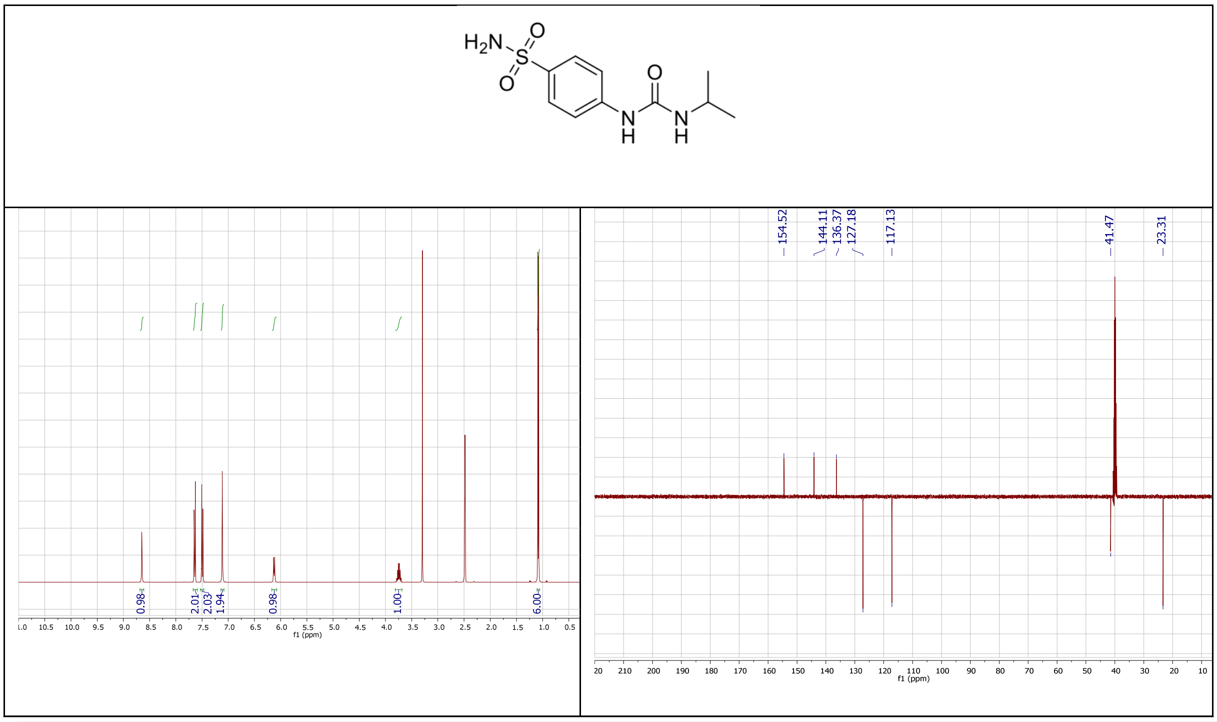

Molecule 3

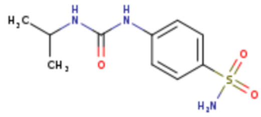

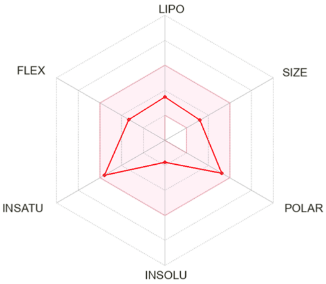

SMILES CC(NC(=O)Nc1ccc(cc1)S(=O)(=O)N)C

Physicochemical Properties	
Formula	C10H15N3O3S
Molecular weight	257.31 g/mol
Num. heavy atoms	17
Num. arom. heavy atoms	6
Fraction Csp3	0.30
Num. rotatable bonds	5
Num. H-bond acceptors	4
Num. H-bond donors	3
Molar Refractivity	64.55
TPSA	109.67 Å²

Lipophilicity	
Log $P_{o/w}$ (iLOGP)	0.75
Log $P_{o/w}$ (XLOGP3)	0.59
Log $P_{o/w}$ (WLOGP)	1.75
Log $P_{o/w}$ (MLOGP)	0.50
Log $P_{o/w}$ (SILICOS-IT)	-0.67
Consensus Log $P_{o/w}$	0.59

Water Solubility	
Log *S* (ESOL)	-1.74
Solubility	4.70e+00 mg/ml ; 1.83e-02 mol/l
Class	Very soluble
Log *S* (Ali)	-2.47
Solubility	8.79e-01 mg/ml ; 3.42e-03 mol/l
Class	Soluble
Log *S* (SILICOS-IT)	-2.84
Solubility	3.69e-01 mg/ml ; 1.44e-03 mol/l
Class	Soluble

Pharmacokinetics	
GI absorption	High
BBB permeant	No
P-gp substrate	No
CYP1A2 inhibitor	No
CYP2C19 inhibitor	No
CYP2C9 inhibitor	No
CYP2D6 inhibitor	No
CYP3A4 inhibitor	No
Log K_p (skin permeation)	-7.45 cm/s

Druglikeness	
Lipinski	Yes; 0 violation
Ghose	Yes
Veber	Yes
Egan	Yes
Muegge	Yes
Bioavailability Score	0.55

Medicinal Chemistry	
PAINS	0 alert
Brenk	0 alert
Leadlikeness	Yes
Synthetic accessibility	2.07

4-(3-Butylureido)benzenesulfonamide (**10**)

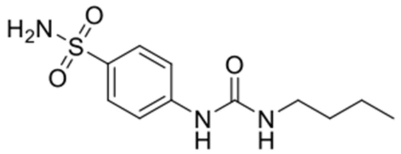

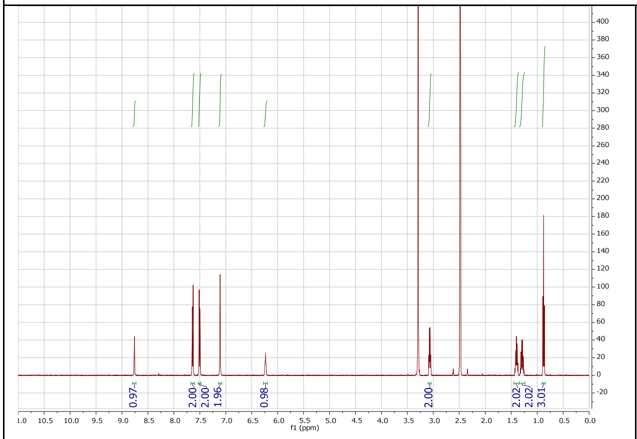

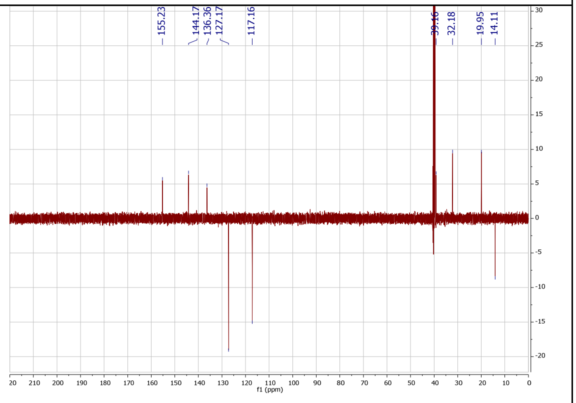

Molecule 4

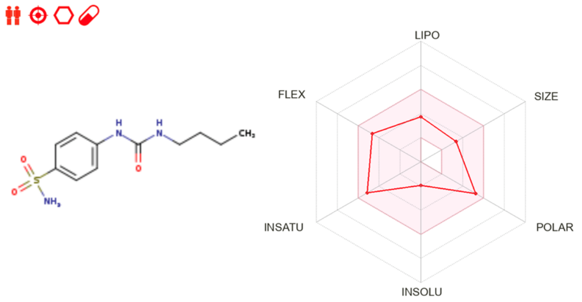

SMILES CCCCNC(=O)Nc1ccc(cc1)S(=O)(=O)N

Physicochemical Properties	
Formula	C11H17N3O3S
Molecular weight	271.34 g/mol
Num. heavy atoms	18
Num. arom. heavy atoms	6
Fraction Csp3	0.36
Num. rotatable bonds	7
Num. H-bond acceptors	4
Num. H-bond donors	3
Molar Refractivity	69.36
TPSA	109.67 Å²

Lipophilicity	
Log $P_{o/w}$ (iLOGP)	1.44
Log $P_{o/w}$ (XLOGP3)	1.04
Log $P_{o/w}$ (WLOGP)	2.15
Log $P_{o/w}$ (MLOGP)	0.79
Log $P_{o/w}$ (SILICOS-IT)	-0.12
Consensus Log $P_{o/w}$	1.06

Water Solubility	
Log *S* (ESOL)	-1.96
Solubility	2.96e+00 mg/ml ; 1.09e-02 mol/l
Class	Very soluble
Log *S* (Ali)	-2.93
Solubility	3.16e-01 mg/ml ; 1.17e-03 mol/l
Class	Soluble
Log *S* (SILICOS-IT)	-3.62
Solubility	6.54e-02 mg/ml ; 2.41e-04 mol/l
Class	Soluble

Pharmacokinetics	
GI absorption	High
BBB permeant	No
P-gp substrate	No
CYP1A2 inhibitor	No
CYP2C19 inhibitor	No
CYP2C9 inhibitor	No
CYP2D6 inhibitor	No
CYP3A4 inhibitor	No
Log K_p (skin permeation)	-7.22 cm/s

Druglikeness	
Lipinski	Yes; 0 violation
Ghose	Yes
Veber	Yes
Egan	Yes
Muegge	Yes
Bioavailability Score	0.55

Medicinal Chemistry	
PAINS	0 alert
Brenk	0 alert
Leadlikeness	Yes
Synthetic accessibility	2.14

4-(3-Hexylureido)benzenesulfonamide (**11**)

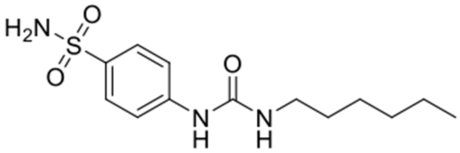

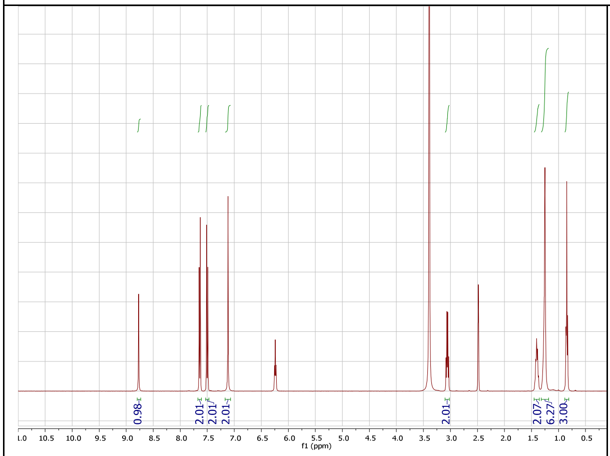

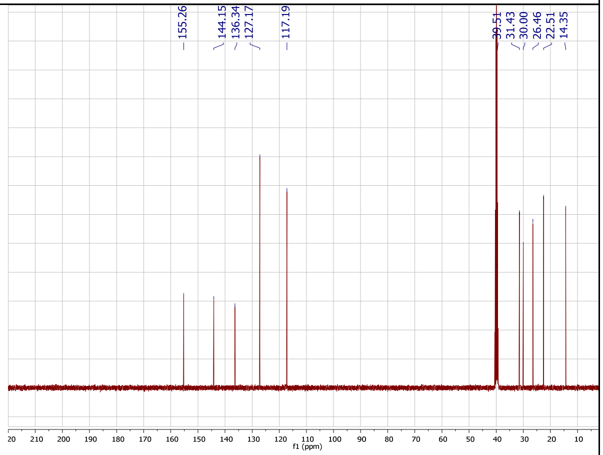

Molecule 3

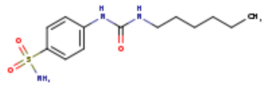

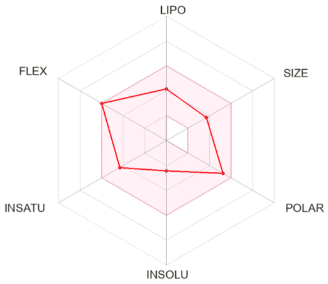

SMILES CCCCCCNC(=O)Nc1ccc(cc1)S(=O)(=O)N

Physicochemical Properties	
Formula	C13H21N3O3S
Molecular weight	299.39 g/mol
Num. heavy atoms	20
Num. arom. heavy atoms	6
Fraction Csp3	0.46
Num. rotatable bonds	9
Num. H-bond acceptors	4
Num. H-bond donors	3
Molar Refractivity	78.97
TPSA	109.67 Å²

Lipophilicity	
Log $P_{o/w}$ (iLOGP)	1.78
Log $P_{o/w}$ (XLOGP3)	1.78
Log $P_{o/w}$ (WLOGP)	2.93
Log $P_{o/w}$ (MLOGP)	1.34
Log $P_{o/w}$ (SILICOS-IT)	0.65
Consensus Log $P_{o/w}$	1.70

Water Solubility	
Log *S* (ESOL)	-2.45
Solubility	1.07e+00 mg/ml ; 3.58e-03 mol/l
Class	Soluble
Log *S* (Ali)	-3.70
Solubility	5.95e-02 mg/ml ; 1.99e-04 mol/l
Class	Soluble
Log *S* (SILICOS-IT)	-4.42
Solubility	1.15e-02 mg/ml ; 3.83e-05 mol/l
Class	Moderately soluble

Pharmacokinetics	
GI absorption	High
BBB permeant	No
P-gp substrate	No
CYP1A2 inhibitor	No
CYP2C19 inhibitor	No
CYP2C9 inhibitor	Yes
CYP2D6 inhibitor	No
CYP3A4 inhibitor	No
Log K_p (skin permeation)	-6.86 cm/s

Druglikeness	
Lipinski	Yes; 0 violation
Ghose	Yes
Veber	Yes
Egan	Yes
Muegge	Yes
Bioavailability Score	0.55

Medicinal Chemistry	
PAINS	0 alert
Brenk	0 alert
Leadlikeness	No; 1 violation: Rotors>7
Synthetic accessibility	2.35

N-[(4-Sulfamoylphenyl)carbamoyl]benzamide (**12**)

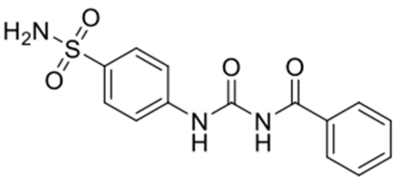

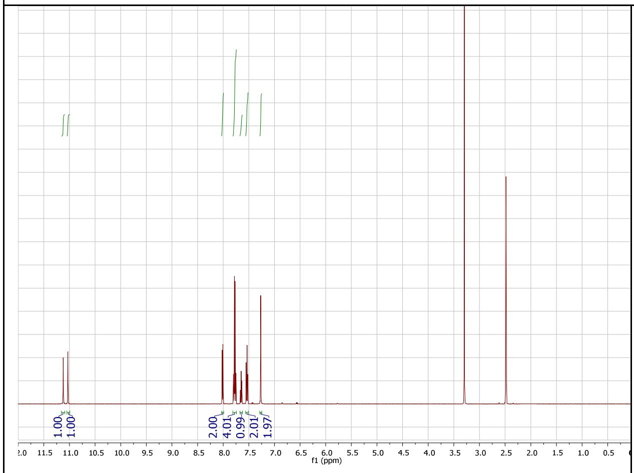

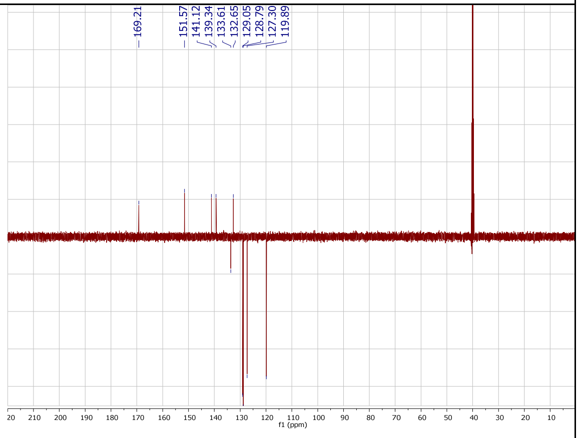

Molecule 4

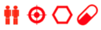

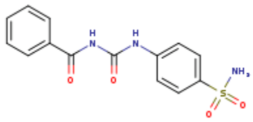

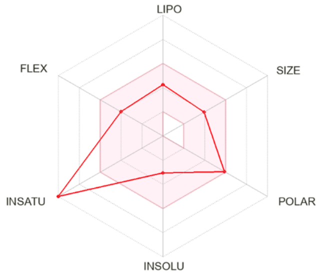

SMILES O=C(Nc1ccc(cc1)S(=O)(=O)N)NC(=O)c1ccccc1

Physicochemical Properties	
Formula	C14H13N3O4S
Molecular weight	319.34 g/mol
Num. heavy atoms	22
Num. arom. heavy atoms	12
Fraction Csp3	0.00
Num. rotatable bonds	6
Num. H-bond acceptors	5
Num. H-bond donors	3
Molar Refractivity	79.85
TPSA	126.74 Å²

Lipophilicity	
Log $P_{o/w}$ (iLOGP)	0.32
Log $P_{o/w}$ (XLOGP3)	1.95
Log $P_{o/w}$ (WLOGP)	2.19
Log $P_{o/w}$ (MLOGP)	1.17
Log $P_{o/w}$ (SILICOS-IT)	-0.17
Consensus Log $P_{o/w}$	1.09

Water Solubility	
Log *S* (ESOL)	-3.06
Solubility	2.81e-01 mg/ml ; 8.79e-04 mol/l
Class	Soluble
Log *S* (Ali)	-4.24
Solubility	1.85e-02 mg/ml ; 5.80e-05 mol/l
Class	Moderately soluble
Log *S* (SILICOS-IT)	-4.46
Solubility	1.10e-02 mg/ml ; 3.46e-05 mol/l
Class	Moderately soluble

Pharmacokinetics	
GI absorption	High
BBB permeant	No
P-gp substrate	No
CYP1A2 inhibitor	No
CYP2C19 inhibitor	No
CYP2C9 inhibitor	No
CYP2D6 inhibitor	No
CYP3A4 inhibitor	No
Log K_p (skin permeation)	-6.86 cm/s

Druglikeness	
Lipinski	Yes; 0 violation
Ghose	Yes
Veber	Yes
Egan	Yes
Muegge	Yes
Bioavailability Score	0.55

Medicinal Chemistry	
PAINS	0 alert
Brenk	0 alert
Leadlikeness	Yes
Synthetic accessibility	2.07

Ethyl 4-[3-(4-sulfamoylphenyl)ureido]benzoate (**13**)

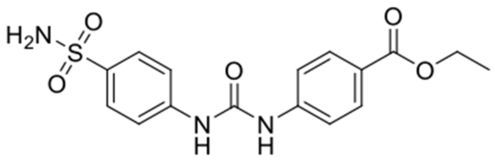

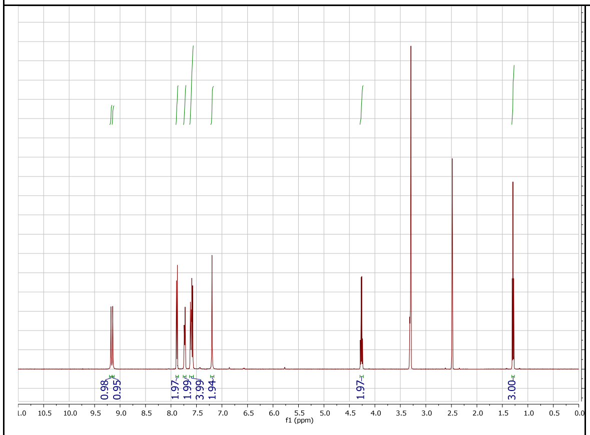

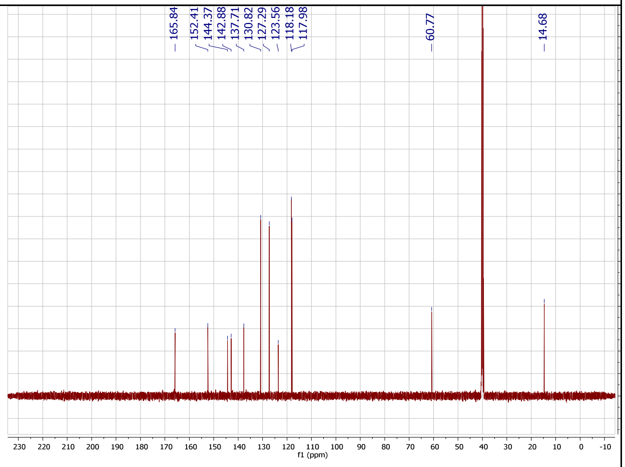

Molecule 5

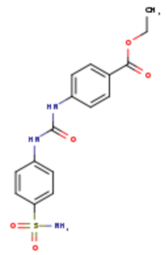

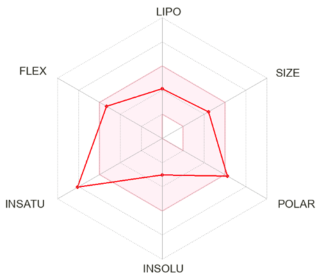

SMILES CCOC(=O)c1ccc(cc1)NC(=O)Nc1ccc(cc1)S(=O)(=O)N

Physicochemical Properties	
Formula	C16H17N3O5S
Molecular weight	363.39 g/mol
Num. heavy atoms	25
Num. arom. heavy atoms	12
Fraction Csp3	0.12
Num. rotatable bonds	8
Num. H-bond acceptors	6
Num. H-bond donors	3
Molar Refractivity	92.24
TPSA	135.97 Å²

Lipophilicity	
Log $P_{o/w}$ (iLOGP)	1.47
Log $P_{o/w}$ (XLOGP3)	1.73
Log $P_{o/w}$ (WLOGP)	2.85
Log $P_{o/w}$ (MLOGP)	1.56
Log $P_{o/w}$ (SILICOS-IT)	0.29
Consensus Log $P_{o/w}$	1.58

Water Solubility	
Log *S* (ESOL)	-3.01
Solubility	3.55e-01 mg/ml ; 9.77e-04 mol/l
Class	Soluble
Log *S* (Ali)	-4.20
Solubility	2.28e-02 mg/ml ; 6.28e-05 mol/l
Class	Moderately soluble
Log *S* (SILICOS-IT)	-4.96
Solubility	3.94e-03 mg/ml ; 1.08e-05 mol/l
Class	Moderately soluble

Pharmacokinetics	
GI absorption	High
BBB permeant	No
P-gp substrate	No
CYP1A2 inhibitor	No
CYP2C19 inhibitor	No
CYP2C9 inhibitor	No
CYP2D6 inhibitor	No
CYP3A4 inhibitor	No
Log K_p (skin permeation)	-7.29 cm/s

Druglikeness	
Lipinski	Yes; 0 violation
Ghose	Yes
Veber	Yes
Egan	No; 1 violation: TPSA>131.6
Muegge	Yes
Bioavailability Score	0.55

Medicinal Chemistry	
PAINS	0 alert
Brenk	0 alert
Leadlikeness	No; 2 violations: MW>350, Rotors>7
Synthetic accessibility	2.43

4-[3-(4-Sulfamoylphenyl)ureido]benzoic acid (**14**)

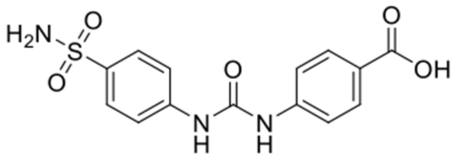

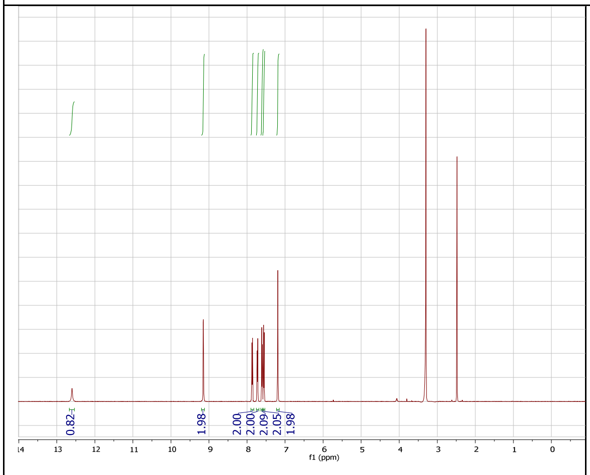

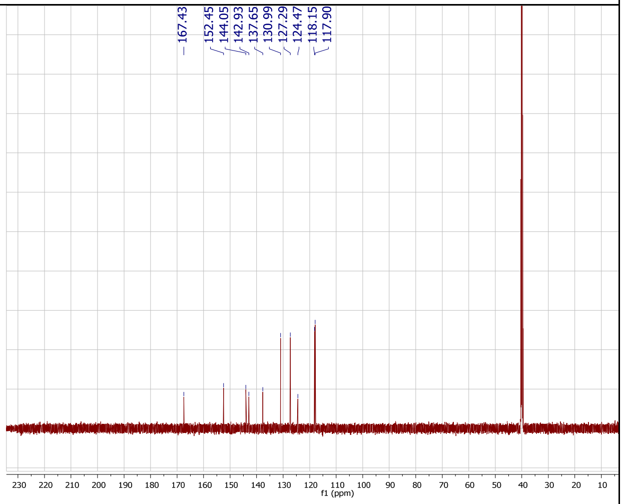

Molecule 1

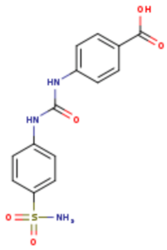

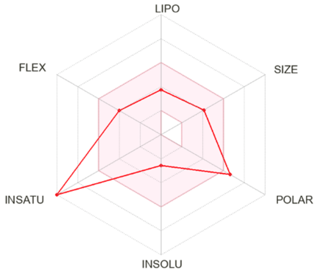

SMILES O=C(Nc1ccc(cc1)S(=O)(=O)N)Nc1ccc(cc1)C(=O)O

Physicochemical Properties	
Formula	C14H13N3O5S
Molecular weight	335.34 g/mol
Num. heavy atoms	23
Num. arom. heavy atoms	12
Fraction Csp3	0.00
Num. rotatable bonds	6
Num. H-bond acceptors	6
Num. H-bond donors	4
Molar Refractivity	83.12
TPSA	146.97 Å²

Lipophilicity	
Log $P_{o/w}$ (iLOGP)	0.68
Log $P_{o/w}$ (XLOGP3)	1.06
Log $P_{o/w}$ (WLOGP)	2.38
Log $P_{o/w}$ (MLOGP)	1.06
Log $P_{o/w}$ (SILICOS-IT)	-0.63
Consensus Log $P_{o/w}$	0.91

Water Solubility	
Log *S* (ESOL)	-2.58
Solubility	8.88e-01 mg/ml ; 2.65e-03 mol/l
Class	Soluble
Log *S* (Ali)	-3.74
Solubility	6.14e-02 mg/ml ; 1.83e-04 mol/l
Class	Soluble
Log *S* (SILICOS-IT)	-3.88
Solubility	4.45e-02 mg/ml ; 1.33e-04 mol/l
Class	Soluble

Pharmacokinetics	
GI absorption	Low
BBB permeant	No
P-gp substrate	No
CYP1A2 inhibitor	No
CYP2C19 inhibitor	No
CYP2C9 inhibitor	No
CYP2D6 inhibitor	No
CYP3A4 inhibitor	No
Log K_p (skin permeation)	-7.59 cm/s

Druglikeness	
Lipinski	Yes; 0 violation
Ghose	Yes
Veber	No; 1 violation: TPSA>140
Egan	No; 1 violation: TPSA>131.6
Muegge	Yes
Bioavailability Score	0.56

Medicinal Chemistry	
PAINS	0 alert
Brenk	0 alert
Leadlikeness	Yes
Synthetic accessibility	2.15

4-[3-(5-Chloro-2-phenoxyphenyl)ureido]benzenesulfonamide (**15**)

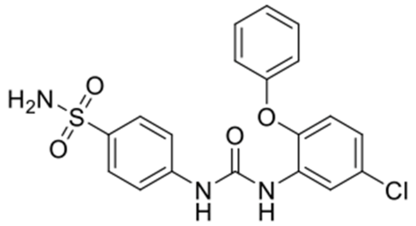

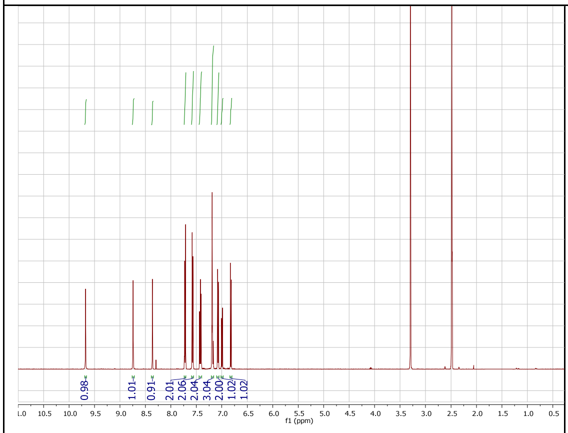

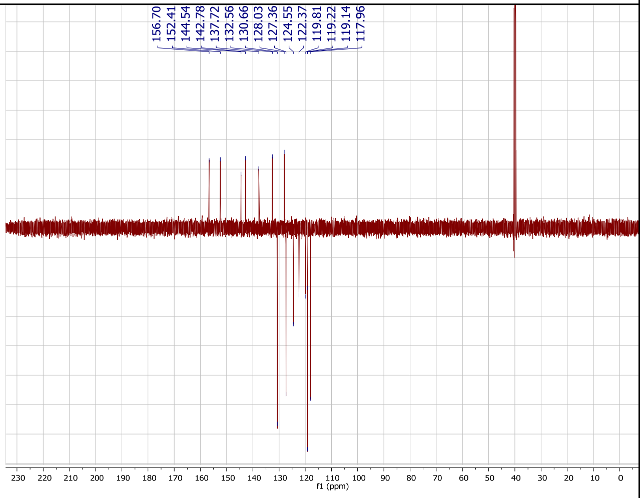

Molecule 4

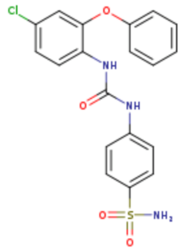

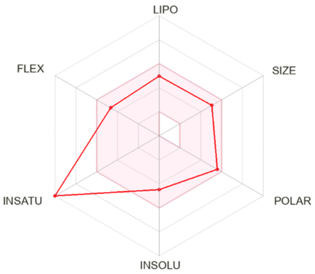

SMILES O=C(Nc1ccc(cc1Oc1ccccc1)Cl)Nc1ccc(cc1)S(=O)(=O)N

Physicochemical Properties	
Formula	C19H16ClN3O4S
Molecular weight	417.87 g/mol
Num. heavy atoms	28
Num. arom. heavy atoms	18
Fraction Csp3	0.00
Num. rotatable bonds	7
Num. H-bond acceptors	5
Num. H-bond donors	3
Molar Refractivity	107.68
TPSA	118.90 Å²

Lipophilicity	
Log $P_{o/w}$ (iLOGP)	1.99
Log $P_{o/w}$ (XLOGP3)	3.21
Log $P_{o/w}$ (WLOGP)	5.12
Log $P_{o/w}$ (MLOGP)	2.87
Log $P_{o/w}$ (SILICOS-IT)	1.69
Consensus Log $P_{o/w}$	2.98

Water Solubility	
Log *S* (ESOL)	-4.47
Solubility	1.43e-02 mg/ml ; 3.41e-05 mol/l
Class	Moderately soluble
Log *S* (Ali)	-5.38
Solubility	1.75e-03 mg/ml ; 4.18e-06 mol/l
Class	Moderately soluble
Log *S* (SILICOS-IT)	-7.31
Solubility	2.04e-05 mg/ml ; 4.88e-08 mol/l
Class	Poorly soluble

Pharmacokinetics	
GI absorption	Low
BBB permeant	No
P-gp substrate	No
CYP1A2 inhibitor	No
CYP2C19 inhibitor	Yes
CYP2C9 inhibitor	Yes
CYP2D6 inhibitor	No
CYP3A4 inhibitor	No
Log K_p (skin permeation)	-6.57 cm/s

Druglikeness	
Lipinski	Yes; 0 violation
Ghose	Yes
Veber	Yes
Egan	Yes
Muegge	Yes
Bioavailability Score	0.55

Medicinal Chemistry	
PAINS	0 alert
Brenk	0 alert
Leadlikeness	No; 1 violation: MW>350
Synthetic accessibility	2.98
